# Supplementary material for: MINT: A toolbox for the analysis of multivariate neural information coding and transmission
Source: PLoS Comput Biol. 2025 Apr 15;21(4):e1012934. doi: 10.1371/journal.pcbi.1012934 (PMC12043240; doi:10.1371/journal.pcbi.1012934)
Supplement: S1 Appendix — Theoretical definitions, descriptions of the available methods in the toolbox, detailed descriptions of the simulation specifications used in the figures as well as supplementary analysis and figures. (PDF) [file pcbi.1012934.s001.pdf]

S1 Appendix: Supplementary Material for "MINT: a toolbox for the analysis of multivariate neural information coding and transmission"

SM1 Comparison with other toolboxes

The following table provides a synthetic comparison of main features of different currently available toolboxes.

Software	Information Measures	Input Data	Signif. Testing	Estimation Methods	Bias corr	Dim Red	Platform
MINT<br>(this paper)	Entropy,<br>MI,<br>TE,<br>Info Breakdown,<br>PID,<br>II,<br>FIT<br>Cond. variants	Discrete<br>Continuous	Non-parametric permutation tests	Binning,<br>Gaussian fit<br>KSG<br>NSB	✓	✓	MATLAB<br>Python wrapper
HOI [1]	Entropy,<br>MI,<br>Redundancy,<br>Synergy,<br>Netw. behavior and encoding metrics	Discrete<br>Continuous	X	Binning,<br>KSG<br>Kernel-based,<br>Gaussian copula	X	X	Python
FRITES<br>[2]	Entropy,<br>MI,<br>TE	Discrete<br>Continuous	Non-parametric, group stats	Binning,<br>Gaussian copula	✓	X	Python
infoTheory<br>[3]	MI<br>(SMGM estimator)	Discrete<br>Continuous	X	X	X	X	MATLAB
Infotheory<br>[4] (Candadai,	Entropy,<br>MI,<br>PID	Discrete<br>Continuous	X	Binning<br>Kernel-based	X	X	Python<br>C++
BROJA-2PID<br>[5]	PID	Discrete	X	X	X	X	Python
Neuroscience Information Theory Toolbox<br>[6]	Entropy,<br>MI,<br>TE,<br>PID,<br>Info transfer,<br>Cond. variants	Discrete<br>Continuous	X	Binning	X	X	MATLAB
dit [7]	Entropy,<br>MI,<br>PID	Discrete	X	Binning<br>KSG<br>Kernel-based	X	X	Python
Gaussian Copula Mutual Information<br>[8]	Entropy,<br>MI	Discrete<br>Continuous	X	Gaussian copula	✓	X	MATLAB<br>Python
Inform<br>[9]	Entropy,<br>MI,<br>TE	Discrete	X	Binning	X	X	C (Python, R, Julia, Mathematica wrappers)
JIDT [10]	Entropy,<br>MI,<br>TE,<br>Cond. variants	Discrete<br>Continuous	Non-parametric	Binning,<br>KSG<br>Kernel-based<br>Gaussian fit	✓	X	Java (Python, MATLAB wrappers)

Software	Information Measures	Input Data	Signif. Testing	Estimation Methods	Bias corr	Dim Red	Platform
MuTE [11]	TE	Continuous	Non-parametric	Binning Gaussian fit Kernel-based	✓	X	MATLAB
ITE [12]	Entropy, MI	Discrete Continuous	X	Kernel-based	X	X	MATLAB Python
Trentool [13]	TE	Continuous	Non-parametric, group stats	KSG Kernel-based	✓	X	MATLAB
Transfer Entropy Toolbox [14]	TE	Spike trains	X	X	X	X	MATLAB
Information Breakdown Toolbox [15]	Entropy, MI, TE Info Breakdown	Discrete Continuous	Non-parametric	Binning, Gaussian fit	✓	X	MATLAB
PyEntropy [16]	Entropy, MI, Max entropy	Discrete Continuous	X	Binning, Shrink estimator	✓	X	Python
STAToolkit [17]	Entropy, MI	Spike trains	Non-parametric	Binning	✓	X	MATLAB (with .mex files)

Table 1: *Comparison of MINT with other existing information-theoretic toolboxes.* Abbreviations of information-theoretic quantities are as follows: FIT=Feature-specific information transfer; II= Intersection Information; MI= Mutual Information; PID=Partial Information Decomposition; TE = Transfer Entropy; KSG = Kraskov-Stögbauer-Grassberger Estimator; NSB = Nemenman-Shafee-Bialek Estimator.

SM2 Description of installation and testing of MINT, and of information theoretic tools implemented in MINT

MINT can be downloaded at the public repository https://github.com/panzerilab/MINT.

Documentation on building and installing the software from source are provided as a README file that specifies the installation requirements, as well as a build file (`BuildMINT.m`) designed to automate the software's compilation process. *Instructions on how a user can test the software on supplied simulated test data* are provided as a folder "How_to_use_MINT" in MINT's public repository containing detailed instructions for testing it on simulated data. We also provide an additional repository (https://github.com/panzerilab/MINT_figures) containing the code that replicates all analyses in all figures, on both real neural data and simulated data. The dataset with CA1 neural data is provided as attachment in in S2 CA1 Data, and the dataset with A1 neural data is provided by the public link https://drum.lib.umd.edu/items/30d43732-7149-4726-a860-0ae3d210b2ae.

For users who prefer conducting their analysis workflows in Python, it is possible to use MINT by setting up the MATLAB Engine API for Python. This requires installing the MATLAB engine library via pip (a package manager for Python), ensuring that the library version matches

the installed version of MATLAB on the system. Detailed installation instructions can be found on the MathWorks website: https://www.mathworks.com/help/matlab/matlab_external/install-the-matlab-engine-for-python.html. To prepare input data, the user can convert Numpy arrays to MATLAB-compatible format using the `matlab.double` conversion function. Optional arguments can be structured as Python dictionaries and used directly. Additionally, the How_to_use_MINT folder includes example Python scripts demonstrating how to initialize the MATLAB engine, format input data and organize the options structure in Python.

MINT provides information theoretic tools to give quantitative answers to questions about information processing when applied to single neurons, population of neurons, or to aggregate neural signals recorded across multiple areas (including LFPs, M/EEG, fMRI). The information processed by the considered neural activity can be about a specific task variable, such as a sensory stimulus, a behavioral output, or about the activity of other neurons or neural populations.

All the information theoretic quantities are functions of the joint probability, sampled across experimental trials, of observing a given value for a set of task variables (e.g. sensory stimuli, movement parameters, behavioral choices) $\boldsymbol{s} \in \boldsymbol{S}$ and of neural responses $(\boldsymbol{r_1}, \dots, \boldsymbol{r_N}) \in \{\boldsymbol{R_1}, \dots, \boldsymbol{R_N}\}$. Each of the variables is indicated with bold font because it can be a multidimensional vector. Importantly, each dimension in the task and neural response variables is assumed to have discrete values, so that the probabilities can be estimated by empirical occurrences. In many cases, neural data will be already discrete in nature (for example, spike counts) and the same applies to some categories of task variables like behavioral choices or identity of the presented stimulus (which experimentally usually fall into a number of discrete categories). In other cases, either task variables or neural responses will be continuous data (e.g. LFPs, etc). These input data will be automatically discretized into a finite number of bins by MINT to perform the information calculations by specifying the number of bins `n_bins` (by default, 3 bins) and the binning strategy `bin_method` (including equi-spaced binning, equi-populated binning, and binning with user-defined bin edges; by default, no binning) as fields within the options input structure `opts`. MINT allows the use of these discretization procedures for all its information theoretic measures, and direct probability distribution sampling after discretization was used for all results in the paper. In addition to binning, MINT offers the option to compress the multi-dimensional neural activity space $(\boldsymbol{r_1}, \dots, \boldsymbol{r_N}) \in \{\boldsymbol{R_1}, \dots, \boldsymbol{R_N}\}$ into a dimensionality reduced representation, obtained with either supervised decoding methods or unsupervised data reduction methods, which can be also discretized and used for information calculations with the direct probability distribution sampling (see Section SM5, Fig. B).

The functions to compute the information quantities in MINT follow a consistent structure (Fig. A). The first input provides the data organized in a cell array. This input should be formatted as {A, B, C, …}. Optionally, the second input can be a cell array of strings called `reqOutputs`, which specifies the quantities to be computed based on the provided datasets,

following the nomenclature of the input format. As a last input the user can provide a structure `opts` that contains optional arguments for the computation.

The outputs of the functions are also implemented in a consistent structure. The first output variable contains cells with the requested information quantities given in `reqOutputs` (in the same order as specified). The second output variable contains cells with the plugin information quantities (i.e., no limited-sampling bias correction) and the last output variable contains the null distribution for each specified information quantity, if the `opts` field `computeNulldist` is set to true. For instance, to compute the limited-sampling bias corrected and the plugin Mutual Information between two populations X1 and X2 (two-dimensional arrays with neurons in the first dimension and trials in the second dimension) the MI function is called as follows:

```
[MI_corr, MI_plugin] = MI({X1, X2},{'I(A;B)'},opts)
```

If the input data for `H.m`, `MI.m`, `cMI.m`, `PID.m` or `II.m` is given as a time series (three-dimensional array, with neuron or brain area ID in the first dimension, time points in the second dimension, and trials in the third dimension), these functions compute the information quantities for each time point and output them as time series.

In the following we list and synthetically describe the information quantities implemented in MINT.

SM2.1 Shannon information

The `MI.m` function computes Shannon Mutual Information between a population of N neurons $\{\boldsymbol{R_1}, \dots, \boldsymbol{R_N}\}$ and a task variable $\boldsymbol{S}$ (such as a sensory stimulus). It is a non-parametric measure that quantifies the full single-trial relationship between $\{\boldsymbol{R_1}, \dots, \boldsymbol{R_N}\}$ and $\boldsymbol{S}$. It is defined as:

$$MI(\boldsymbol{S}; \boldsymbol{R_1}, \dots, \boldsymbol{R_N}) = \sum_{s,r_1,\dots,r_N} p(\boldsymbol{s}, \boldsymbol{r_1}, \dots, \boldsymbol{r_N}) \log_2 \left(\frac{p(s,r_1,\dots,r_N)}{p(s)p(r_1,\dots,r_N)}\right) \quad (1)$$

where $p(\boldsymbol{s}, \boldsymbol{r_1}, \dots, \boldsymbol{r_N})$ is the joint probability, sampled across experimental trials, of observing stimulus value $s \in S$ and the neural responses $(\boldsymbol{r_1}, \dots, \boldsymbol{r_N}) \in \{\boldsymbol{R_1}, \dots, \boldsymbol{R_N}\}$, and $p(\boldsymbol{s})$ and $p(\boldsymbol{r_1}, \dots, \boldsymbol{r_N})$ are the marginal probabilities of observing s and $(\boldsymbol{r_1}, \dots, \boldsymbol{r_N})$, respectively. The sum in Eq. (1) spans all possible events. $MI(\boldsymbol{S}; \boldsymbol{R_1}, \dots, \boldsymbol{R_N})$ is non-negative and is zero if and only if $\{\boldsymbol{R_1}, \dots, \boldsymbol{R_N}\}$ and $\boldsymbol{S}$ are independent. To compute the Shannon Mutual Information of two variables, `reqOutputs` has to be defined as `'I(A;B)'`. Moreover, MINT also allows to compute the mutual information between $\boldsymbol{S}$ and $\{\boldsymbol{R_1}, \dots, \boldsymbol{R_N}\}$ conditioned on the activity of another population of M neurons $\{\boldsymbol{R'_1}, \dots, \boldsymbol{R'_M}\}$ or another stimulus feature $\boldsymbol{S'}$ (function `cMI.m`).

SM2.2 Information Breakdown

The `MI.m` function can also compute measures that quantify how interactions between neurons contribute to the encoding of $\boldsymbol{S}$. The desired quantities can be passed to the function as specific strings within the `reqOutputs` cell array input. These quantities include the redundancy-

synergy index $\boldsymbol{RSI}$ (`reqOutputs` defined as `'RSI(A;B)'`), which is defined as the difference between the population information and the sum of single-neuron stimulus information quantifying the overall contribution of interactions to population encoding. Additionally, the function can return Information Breakdown terms [18] quantifying how pairwise correlations contribute to the redundancy-synergy index [19]. These terms include the signal-similarity $\boldsymbol{I_{sig-sim}}$ (contribution of the similarity across neurons of trial-averaged responses to different stimuli, `reqOutputs` defined as `'Iss(A)'`), the stimulus-independent correlations $\boldsymbol{I_{cor-ind}}$ (contribution of the interplay between the signs of signal similarity and of noise correlations, `reqOutputs` defined as `'Ici(A;B)'`), and the stimulus-dependent correlations $\boldsymbol{I_{cor-dep}}$ (quantifying how much information is gained by the stimulus-modulation of noise correlations, `reqOutputs` defined as `'Icd(A;B)'`).

SM2.3 Partial Information Decomposition (PID)

The `PID.m` function computes PID components. PID decomposes the information jointly carried by a set of source variables (for us, a set of simultaneously recorded neurons; the first N variables in the `inputs` cell array) about a target *S* (for us, a sensory stimulus or a behavioral choice; the last variable in the `inputs` cell array) into non-negative components that capture information about the target that is either redundantly encoded across sources, uniquely encoded by a single source or synergistically encoded by the combination of sources. For more than two source variables such components can also represent combinations of redundancy, synergy and unique information. In the case of two source variables, once the individual Mutual Information between the target and each source and the joint Mutual Information between the targeted and the two sources, and one of the PID components (e.g. redundancy) are all computed, algebraic linear relationships (derived from PID "lattices") allow to compute all remaining PID components. Thus, a PID with two sources is defined by the choice of a specific redundancy measure. The desired PID components can be passed to the function as strings within the `reqOutputs` cell array input (`'Red'` for redundancy, `'Syn'` for synergy, `'Unq1'` and `'Unq2'` for the unique information carried by the first or the second input source, respectively). For more than two variables, defining a redundancy measure is enough to compute each PID component as a linear combination between redundancy terms (the output name `'PID_atoms'` provides all PID components). Therefore, different methods to decompose information differ and are defined by the measure of redundancy they use (which can be specified as the `redundancy_measure` field in the `opts` structure). In MINT we implemented three possible measures of PID redundancy, which are very popular and respect the so called pairwise marginal property (redundancy is invariant for distributions preserving the pairwise marginals between each source and the target).

SM2.3.1 I_{min}

We implemented Williams and Beer's PID original redundancy measure called I_{min}. This measure [70] quantifies the information redundantly encoded about $\boldsymbol{S}$ across N source variables $\{\boldsymbol{R_1},...,\boldsymbol{R_N}\}$ as:

$$Red(\boldsymbol{S}: \boldsymbol{R_1}; \boldsymbol{R_2}; \dots; \boldsymbol{R_N}) = I_{min}(\boldsymbol{S}: \boldsymbol{R_1}; \boldsymbol{R_2}; \dots; \boldsymbol{R_N}) = \sum_{\boldsymbol{s} \in \boldsymbol{S}} p(\boldsymbol{s}) \min_{R_i \in \{\boldsymbol{R_1}, \boldsymbol{R_2}, \dots, \boldsymbol{R_N}\}} I(\boldsymbol{S} = s; R_i) \quad (2)$$

where $I(\boldsymbol{S} = \boldsymbol{s}; \boldsymbol{R_i})$ represents the specific information that source variable $\boldsymbol{R_i}$ provides about a specific value $\boldsymbol{s}$ of the target variable $\boldsymbol{S}$. I_{min} captures redundancy as the similarity across the source variables $\boldsymbol{R_i}$ in distinguishing individual values of $\boldsymbol{S}$.

SM2.3.2 Minimum Mutual Information (I_{MMI})

MINT also implements the PID based on the redundancy measure introduced in [73], called I_{MMI}. This measure quantifies the information redundantly encoded about $\boldsymbol{S}$ across N source variables $\{\boldsymbol{R_1}, \dots, \boldsymbol{R_N}\}$ as:

$$Red(\boldsymbol{S}: \boldsymbol{R_1}; \boldsymbol{R_2}; \dots; \boldsymbol{R_N}) = I_{MMI}(\boldsymbol{S}: \boldsymbol{R_1}; \boldsymbol{R_2}; \dots; \boldsymbol{R_N}) = \min_{\boldsymbol{R_i} \in \{\boldsymbol{R_1}, \boldsymbol{R_2}, \dots, \boldsymbol{R_N}\}} I(\boldsymbol{S}; \boldsymbol{R_i}) \quad (3)$$

Therefore I_{MMI} captures redundancy as the minimum amount of information encoded about $\boldsymbol{S}$ by any of the source variables. When applied to Gaussian variables, its results coincide with the ones calculated through the I_{min} (SM2.3.1) and the BROJA definitions (described below, SM2.3.3).

SM2.3.3 BROJA

Finally, MINT implements the PID based on the redundancy measure termed BROJA [72], which defines redundancy about $\boldsymbol{S}$ between two source variables $\boldsymbol{R_1}$ and $\boldsymbol{R_2}$ as the result of a constrained optimization problem:

$$Red(\boldsymbol{S}: \boldsymbol{R_1}; \boldsymbol{R_2}) = I_{BROJA}(\boldsymbol{S}: \boldsymbol{R_1}; \boldsymbol{R_2}) = MI(\boldsymbol{S}; \boldsymbol{R_1}) + MI(\boldsymbol{S}; \boldsymbol{R_2}) - \min_{q \in \Delta_P} \left(MI_q(\boldsymbol{S}; \boldsymbol{R_1}, \boldsymbol{R_2}) \right) \quad (4)$$

where the minimum of the Mutual Information is taken over the space $\boldsymbol{\Delta_P}$ of distributions preserving pairwise marginals between individual source variables and the target variable. BROJA's advantage is that it is additive for independent systems of sources and targets, however it is only defined for two source variables. The numerical calculation of Eq. (4) is performed by MINT through the conic optimization Embedded Conic Solver (ECOS) algorithm [5]. The use of ECOS requires the installation of a C library which can be implemented by either compiling the C source code with a MATLAB-compatible C Compiler (using MINT's `BuildMINT.m` function) or copying locally the precompiled binaries that MINT provides for Linux, Windows and macOS.

SM2.4 Information transmission measures

MINT provides a number of measures to quantify the overall or feature-specific directed information transmitted from a putative sender region $\boldsymbol{X}$ to a receiver region $\boldsymbol{Y}$ from which neural activity was simultaneously recorded. These measures are all based on the Wiener-Granger causality principle which states that a region $\boldsymbol{X}$ causally influences a region $\boldsymbol{Y}$ if the past state of $\boldsymbol{X}$ predicts the present state of $\boldsymbol{Y}$ at time t beyond what can be predicted by the past state of $\boldsymbol{Y}$.

SM 2.4.1 Transfer Entropy (TE)

Transfer Entropy from $\boldsymbol{X}$ to $\boldsymbol{Y}$, $TE(\boldsymbol{X} \rightarrow \boldsymbol{Y})$ [74], measures the overall information transmitted from region $\boldsymbol{X}$ to region $\boldsymbol{Y}$ and can be computed using the `TE.m` function in the toolbox. $TE(\boldsymbol{X} \rightarrow \boldsymbol{Y})$ is defined as the conditional Mutual Information between the past activity of the sender region $\boldsymbol{X}_{past}$ and the present activity of the receiver region $\boldsymbol{Y}_{pres}$ given the past of the receiver $\boldsymbol{Y}_{past}$. Moreover, MINT also allows to compute TE from $\boldsymbol{X}$ to $\boldsymbol{Y}$ conditioned on the activity of a third node $\boldsymbol{Z}$ (function `cTE.m`; $\boldsymbol{Z}$ can, in principle, also be the multivariate activity of a set of regions).

Although in the example computations of TE provided in Fig. 4, and in supplementary Figs. E and F we computed the present of Y at a single time point t and the past of $\boldsymbol{X}$ and of $\boldsymbol{Y}$ at individual time points lagged by a delay Δt, MINT also allows computing information transfer measures for present and/or past activity as multidimensional variables, potentially spanning several time points. The past-time embedding is specified by the parameters `opts 'tpres'` and `opts 'tau'`. The parameter `'tpres'` specifies the present timepoint in the calculation. The parameter `'tau'` defines the delay (or an arbitrary set of delay numbers if more past points are to be considered) relative to `'tpres'`, expressed as an integer number indicating how many timepoints back the past timepoint is set. These parameters can be specified individually for $\boldsymbol{X}$ and $\boldsymbol{Y}$, allowing the function to use different timepoints for the present and past for each of $\boldsymbol{X}$ and $\boldsymbol{Y}$. This allows easy implementation of various embedding techniques such as those described in [20]. Because of the problem of data dimensionality (the bias can be corrected well if the product of the number of bins for the past of $\boldsymbol{X}$ and the present of $\boldsymbol{X}$ and $\boldsymbol{Y}$ is several times smaller than the number of time samples), many studies (e.g. [21]) use only one time point compute the past of $\boldsymbol{X}$ and $\boldsymbol{Y}$. Some studies [13, 22] have proposed to use a delay of one time-step for the past of the putative receiver $\boldsymbol{Y}$, to be as conservative as possible when conditioning away the information of the putative receiver $\boldsymbol{Y}$. Our own recommendation is to consider and plot a wide range of delays, as we do in our analyses (see e.g. Fig. F panel B), to get a better feeling of the robustness of the results as a function of these parameters.

The string to define in `reqOutputs` to compute Transfer Entropy from the data in the first to the data in the second element of the `input` cell is `'TE(A->B)'`. In addition, a second output can be requested with `'TE(B->A)'`, in order to reduce computational effort and exploring data more efficiently by calculating both directions of Transfer Entropy with a single function call.

SM 2.4.2 Feature-specific information transfer (FIT)

Feature specific information transfer from $\boldsymbol{X}$ to $\boldsymbol{Y}$ about a stimulus feature S $FIT(\boldsymbol{S} \rightarrow \boldsymbol{X} \rightarrow \boldsymbol{Y})$ measures the information transmitted from $\boldsymbol{X}$ to $\boldsymbol{Y}$ about a specific feature $\boldsymbol{S}$ and can be computed using the `FIT.m` function in the toolbox. $FIT(\boldsymbol{S} \rightarrow \boldsymbol{X} \rightarrow \boldsymbol{Y})$ is defined as the minimum between two PID terms with similar but slightly different interpretations. The first term is the information about $\boldsymbol{S}$ that is redundant between $\boldsymbol{X}_{past}$ and $\boldsymbol{Y}_{pres}$, and is unique to

any information encoded by $\boldsymbol{Y}_{past}$. The second term is the information about the present activity of the receiver $\boldsymbol{Y}_{pres}$ that is redundant between $\boldsymbol{X}_{past}$ and $\boldsymbol{S}$, and its unique to any information encoded by $\boldsymbol{Y}_{past}$. To guarantee the nonnegativity of FIT, both terms are computed using the I_{min} measure [23]. Minimizing between the two terms ensures key FIT properties, including that it is upper bounded by the feature information encoded by the past of region X $I(\boldsymbol{S};\ \boldsymbol{X}_{past})$, the one encoded by the present of region $\boldsymbol{Y}$ $I\left(\boldsymbol{S};\boldsymbol{Y}_{pres}\right)$ and by the overall information transmitted from $\boldsymbol{X}$ to $\boldsymbol{Y}$ $TE(\boldsymbol{X} \rightarrow \boldsymbol{Y})$. Moreover, MINT allows for the computation of conditional FIT (cFIT, using the `cFIT.m` function), to remove from $FIT(\boldsymbol{S} \rightarrow \boldsymbol{X} \rightarrow \boldsymbol{Y})$ the component potentially routed through the past activity of a third recorded region $\boldsymbol{Z}$ ($\boldsymbol{Z}$ can, in principle, also be the multivariate activity of a set of regions). $cFIT(\boldsymbol{S} \rightarrow \boldsymbol{X} \rightarrow \boldsymbol{Y}|\boldsymbol{Z})$ is defined as $FIT(\boldsymbol{S} \rightarrow \boldsymbol{X} \rightarrow \boldsymbol{Y})$ minus a term capturing feature information transmitted from $\boldsymbol{X}$ to $\boldsymbol{Y}$ that is also redundantly encoded by the past of $\boldsymbol{Z}$ [23].

Similar to TE, the past-time embedding is specified by the parameters `opts 'tpres'` and `opts 'tau'` to specify the timepoints taken as present and past for the computation. Our own recommendation is to consider and plot a wide range of delays, as we do in our analyses (see e.g. Fig. F panel B), to get a better feeling of the robustness of the results as a function of these parameters.

Similar to the TE function, the FIT function can compute bidirectional information transfer about a target by specifying `'FIT(A->B;S)'` and `'FIT(B->A;S)'` in the `reqOutputs` cell array. The same applies to cFIT in `cFit.m` (specifying `'cFIT(A->B;S|C)'` and `'cFIT(B->A;S|C)'` in the `reqOutputs` cell array).

SM2.5 Intersection Information (II)

MINT implements also Intersection Information (II, computed using the `II.m` function), a measure quantifying the amount of sensory information encoded by neural activity that is used to inform behavioral choices. Intersection Information [24-26] quantifies the part of information in neural responses that is common to both stimulus and choice information. Intersection Information is computed as the minimum between two PID terms with similar but slightly different interpretations. The first term is the information about choice $\boldsymbol{C}$ redundant between stimulus $\boldsymbol{S}$ and neural response $\boldsymbol{R}$. The second term is the information about stimulus $\boldsymbol{S}$ redundant between choice $\boldsymbol{C}$ and neural response $\boldsymbol{R}$. By default, these terms are computed using the BROJA redundancy measure. Minimizing between the two terms ensures that Intersection Information satisfies key properties that would be expected from a measure with this interpretation, including that independent $\boldsymbol{S}$ and $\boldsymbol{R}$ imply null intersection information, that Intersection Information satisfies the data processing inequality, and that Intersection Information is upper bounded by both $MI(\boldsymbol{R};\boldsymbol{S})$ and $MI(\boldsymbol{R};\boldsymbol{C})$.
To compute the Intersection Information as the amount of information present in input data A, encoded in input data B that is also present in input data C, `reqOutputs` can be defined as `'II(A,B,C)'`.

SM3 Information estimators and limited-sampling bias corrections

MINT implements several types of information estimations. Methods based on estimating probabilities of discrete data or through data discretization include the plug-in method and its bias corrections (Shuffle, QE, Shuffle QE, Panzeri-Treves, Ish and BUB). Methods not requiring discretization of data to estimate probabilities include the NBS and KSG methods.

MINT implements limited-sampling bias corrections of information theoretic quantities. For Shannon Entropy, Mutual Information and for the Information Breakdown terms, bias-corrected estimates are computed separately for each quantity. For the PID calculation, bias-corrected estimates can be obtained either by correcting each PID atom individually, or alternatively by correcting for the bias first the individual and joint mutual information term and one of the PID terms (e.g. redundancy or synergy), and then by correcting for the bias of the other PID components by using algebraic relationships derived from PID "lattices" (see SM 3.7). By default, no bias correction is computed (plugin information quantities).

SM3.1 Direct estimator for discrete or discretized data

The direct estimator (or direct method) consists in the direct estimation from the probabilities of discrete data estimated as empirical occurrences, which are then plugged into the appropriate information equations. This method is known to suffer from a downward limited-sampling bias for entropies and of an upward limited-sampling bias for information quantities, which can be corrected with some of the limited bias-correction methods listed below. The estimator computed with the direct method just plugin in the empirical probabilities without any bias correction is referred to as the plugin or uncorrected or naïve estimator. The estimator obtained from the plugin after bias correction is referred to as the bias-corrected estimator. Bias corrections available for the direct method are listed below.

SM 3.1.1 Shuffle Limited-sampling bias correction

When this bias correction option is called (setting the `bias` field in the options structure `opts` to `'shuffSub'`), the bias is estimated by computing the information values after destroying all genuine stimulus information by randomly permuting the stimulus-neural response association in each trial [27, 28]\, and then it is subtracted out from the original estimate to provide the bias-corrected estimate. MINT allows to subtract these bias estimates from all implemented information theoretic quantities. A non-zero integer in the field `shuff` of the options structure `opts` specifies the number of shuffles to be performed and averaged over to obtain this estimate (by default, 20 shuffles).

SM 3.1.2 QE correction

The Quadratic Extrapolation (QE) procedure [29] (setting the `bias` field in the options structure `opts` to `'qe'`) assumes that the estimation is performed in a regime with large numbers of trials and approximates the bias of the information quantities as a second-order

expansions in the inverse of the number of available trials [30]. This procedure first re-computes the information from fractions (halves and quarters) of the data available and then fits the dependence of information estimates on the inverse number of trials to a quadratic function. This quadratic fit is then used to estimate the bias-corrected information value as the value that would be obtained with the quadratic scaling law if an infinite number of trials were available (i.e., the intercept term of the fit). MINT allows the use of this bias correction with all its information theoretic measures. Field `xtrp` in the options structure `opts` specifies the number of repetitions of the extrapolation procedure. The function performs the specified number of extrapolations and calculates the final corrected value as the mean of the estimates (by default, 10). Note that MINT allows the option to perform a linear (rather than quadratic) extrapolation, which uses only halves and not quarters of the data. This may be convenient when very small datasets are available and the division into quarters is problematic.

SM 3.1.3 Shuffle-QE correction

MINT also implements a bias correction combining both Shuffle and QE (setting the `bias` field in the options structure `opts` to `'qe_shuffSub'`). This procedure first computes both the original and the shuffled values, performs QE on both as explained above and then computes the unbiased estimate by subtracting out the QE-corrected shuffled value from the QE-corrected non-shuffled value. The parameters `shuff` and `xtrp` can be set in the options `opts` structure as mentioned above.

SM 3.1.4 Panzeri-Treves correction

This correction technique analytically approximates the linear term of the bias expansion in the inverse of the number of available trials, which is then subtracted from the measured information to obtain bias-corrected information values [27, 30]. The estimation of the bias depends only on the number of response bins with a non-zero probability of being observed, which is estimated using a Bayes approach. It is available only to Shannon Entropy and Mutual Information (setting the `bias` field in the options structure `opts` to `'pt'`) but not to PID-based quantities.

SM 3.1.5 Ish bias reduction procedure for multi-dimensional data

The I_{sh} procedure is relevant for the reduction of the bias of the information about a task variable (say stimulus S) carried by the joint observation of a multivariate neural response with dimension N (e.g. the activity of N neurons). It adds and subtracts to the definition of Mutual Information two entropy terms which have equal asymptotic value (in case of exact sampling of the probabilities with an asymptotically large number of trials). For limited number of trials, the difference between these two terms provides a negative contribution to the Mutual Information bias. Thus, I_{sh} has a considerably smaller bias (though larger variance) than the direct estimate of Mutual Information from Eq. (1). Another interesting property of I_{sh} is that it typically has a negative bias, whereas direct estimates of Mutual Information typically have a positive bias. Thus, the joint calculation of Mutual Information from Eq (1) and I_{sh} allows the estimation of upper and lower bounds to the real information values. This procedure can be applied to the Mutual Information and to the Information Breakdown (where it allows

computation of upper and lower bounds of some terms) (setting the `bias` field in the options structure `opts` to `'shuffCorr'`). It can be combined with the QE and the shuffle-subtraction corrections.

SM 3.1.6 Best Universal Bound procedure (BUB)

This method developed by [31] expresses the information estimation as a polynomial approximation problem, allowing the computation of 'Best Universal Bounds' on the information bias and variance (setting the `bias` field in the options structure `opts` to `'bub'`). Its results depend on the selection of a parameter k_{max} related to degrees of freedom and specified in the field `k_max` in the options structure `opts` (by default, 10).

SM 3.1.7 Options for PID bias

For QE, shuffle subtraction, and shuffle-QE subtraction applied to PID with two source variables, two bias-correction options are available. The first option is to correct for the bias, with the chosen bias correction procedure, each PID term individually. The second option is to correct for the bias in a way that respects the linear relationships between the PID terms and Shannon information quantities derived from the so called PID lattices. This is done by correcting for the bias the individual and joint mutual information term and one PID component of choice, and then correct for the bias the other PID components by using the algebraic relationships derived from PID "lattices". The chosen PID atom can be specified with field `chosen_atom` in the options structure `opts` (by default, synergy). This option is implemented by default (setting the `opts` field `'pidConstrained'` to true). If one chooses not to, each PID atom is corrected individually.

SM3.2 Estimators not requiring discrete or discretized data

SM 3.2.1 Kraskov-Stögbauer-Grassberger (KSG) estimator

The KSG estimator uses the Kozachenko-Leonenko kth-nearest-neighbor entropy estimator to find structures in the underlying probability distribution [32]. The estimator is included in the toolbox using scripts from the improved version of KSG implemented in [33], which was shown to work very well for real-valued data with am most a handful of dimensions. By varying the parameter k which determines the nearest-neighbor statistics scale, one can also change the scale in which the algorithm looks for the underlying structure. The method can be used by setting the `bias` field in the options structure `opts` to `'ksg'` and the parameter k (by default, 6) can be changed by specifying the `k_ksg` field of the `opts` structure.

SM 3.2.2 Nemenman-Shafee-Bialek (NSB) estimator

The NSB estimator is a Bayesian entropy estimator that is designed to work on prior distributions of the stimulus-response distributions that are almost uniform in their expected entropies [34]. This means that the entropy estimate is not strongly biased by the prior assumptions. The method does not require free parameters to be inputted and can be used by setting the `bias` field in the options structure `opts` to `'nsb'`.

SM4 Hierarchical permutations for testing statistical significance and the impact of correlations among different data dimensions in information coding

SM4.1 Data shuffling

Shuffling neural data is a useful tool to test hypothesis and gain insights in the information encoding structure of neural populations and how information is transferred. The `hShuffle.m` function provides a range of hierarchical data shuffling methods, allowing for disruption of neural correlations, temporal patterns or stimulus information. Each data feature (a neural activity dimension or a task variable) can be shuffled either unconditionally on any other variable or conditionally on the values of other variables. For example, neural responses can be shuffled unconditionally on any other variable to destroy the information they carry about the stimulus (e.g., to shuffle the first two input variables across trials the `reqOutputs` can be defined as `'AB'` and the `opts` field `'dim_shuffle'` is set to `'Trials'`). Alternatively, neural responses of different neurons can be shuffled conditional on the stimulus values to provide surrogate data that preserve single-neuron stimulus information but that destroy noise correlations (correlations at fixed stimulus between different neurons). Shuffling the neural responses across trials with the same stimulus but keeping the position of each timepoint fixed provides surrogate data that keep time-resolved stimulus information of single neurons but disrupt across-time correlations. For example, to shuffle the first input variable conditioned on the second and third input variables the `reqOutputs` cell can be defined as `'A_BC'` (the variable(s) to condition the shuffling on are specified after the underscore) and the `opts` field `'dim_shuffle'` is set to `'Trials'`.

Shuffled data are either used within a given function (e.g. `MI.m` or `FIT.m`) to output null hypothesis values, or to be separately provided as input to any function in MINT to construct user-defined non-parametric null distribution to empirically estimate the p-value of measures computed from the original data.

Furthermore, MINT enables efficient computation of group-level averaged shuffled quantities by recombining shuffled information values across experiment repetitions using the `create_nullDist_groupLevel` function. This function accepts measures calculated from M independent data shuffles across N experiment repetitions and outputs K distinct realizations of the permuted group average.

SM4.2 Cluster permutation for multiple comparison correction

It is often important to detect significant information encoded or transmitted across data points that are correlated due to physical proximity (e.g., in space and time, or time and communication delay). MINT implements the rigorous detection of clusters of adjacent significant information values via cluster permutation tests [2] (`clusterStatistics.m` function). The `clusterStatistics.m` function takes as input a matrix of information values computed from the original data across adjacent space and time points and a set of M analogous matrices obtained from shuffled data. The function computes a cluster-forming

threshold as a specified percentile `clusterPercentilThreshold` (provided as input) of the shuffled information values. This threshold is calculated either by pooling shuffled values across all samples (when the input `pool` parameter is set to 1) or independently for each sample (when `pool` is set to 0). The latter option provides less statistical power but is recommended when shuffled information is non-stationary over space and time. The procedure then identifies clusters in both the original and shuffled data by connecting adjacent information values that surpass the cluster-forming threshold and computes the mass of each cluster as the sum of its information values. A cluster-level null distribution is created by taking the maximum cluster mass from each shuffled dataset. Finally, clusters in the original dataset that exceed a specified percentile `significanceThreshold` (provided as input) of this null distribution are classed as significant.

SM5 Interfacing information calculations with dimensionality reduction methods

To allow information analysis on datasets with a high number of dimensions, we offer several possibles wrappers that integrate tools or dimensionality reduction in a way that permits the calculation of information measures on processed variables with less dimensionality.

SM5.1 Interfacing information calculation with supervised dimensionality-reduction method

Here we describe which supervised dimensionality reduction algorithm we implemented in MINT and we interfaced then with the information theoretic algorithms.

Our routines take input data in the form of a cell array with neural responses $(\boldsymbol{r_1}, \ldots, \boldsymbol{r_N}) \in \{\boldsymbol{R_1}, \ldots, \boldsymbol{R_N}\}$ across all trials in the first element and the task variables $\boldsymbol{s} \in \boldsymbol{S}$ (e.g. sensory stimuli, movement parameters, behavioral choices) across all trials in the second element. It returns an array across all trials of dimensionality-reduced representation $\hat{\boldsymbol{r}}$ of $(\boldsymbol{r_1}, \ldots, \boldsymbol{r_N})$. The dimensionality-reduced representation $\widehat{\boldsymbol{R}}$ is computed by cross-validated decoding of the behavioral variables $\boldsymbol{s}$ given the neural data, so that the representation $\hat{\boldsymbol{r}}$ for each trial is computed from trials held out from the decoder's training process. The reduced neural representation data are then fed as neural data input to the information calculation routines.

The reduced representation $\widehat{\boldsymbol{R}}$ obtained through the supervised decoding method can be understood as a representation of the neural activity data which is lower dimensional (typically, one dimensional) but that still captures efficiently the information about $\boldsymbol{S}$ provided by the joint neural responses. The reduced representation $\widehat{\boldsymbol{R}}$ can take form of the value of the behavioral variable decoded as most likely from neural activity (which can be directly fed to the information calculation routines, an approach that is equivalent to computing information from the confusion matrix of the decoder), or the posterior probability of the task variable value given the considered neural activity (which can then be binned and fed to the information calculation routines).

Mutual Information between the stimulus and the reduced neural representation $\hat{\boldsymbol{R}}$, and Intersection information between stimulus, choices, and the reduced neural representation $\hat{\boldsymbol{R}}$ are obtained by simply using the reduced neural representation $\hat{\boldsymbol{R}}$ instead of the actual neural activity $\boldsymbol{R}$ into the corresponding equations defined in sections SM 2.1 and SM2.5. Importantly, both the Mutual and the Intersection Information satisfy the data processing inequality, which implies that the values obtained through the reduced neural representation are lower bounds to the values of the full, unreduced, neural activity representation.

Supervised models implemented in MINT are:

SM5.1.1 Support Vector Machines (SVM)

Support vector machines (SVM) are supervised machine learning methods that find the optimal hyperplane/s in the data space (in our case neural data $(\boldsymbol{r_1}, \dots, \boldsymbol{r_N})$) to classify the labels (in our case $\boldsymbol{s}$). MINT provides a function `svm_wrapper.m` that trains and tests the SVM with either linear or radial basis function (RBF) kernel, using either `fitcsvm.m` (for binary labels; Statistics and Machine Learning Toolbox in MATLAB), `fitcecoc.m` (for multiclass labels; Statistics and Machine Learning Toolbox in MATLAB) or the libsvm toolbox [35] as the underlying SVM implementation. The first element of the output cell array is the lower dimensional representation $\hat{\boldsymbol{R}}$ as an array of the cross-validated predicted labels across trials. The second element of the output cell array is the representation $\hat{\boldsymbol{R}}$ as an array of cross-validated posterior probabilities of $\boldsymbol{s}$ given the neural responses in that trial. The function can also output the weights of the decoding model for the linear SVM (which can be used e.g. to calculate the angle between boundaries as in Fig. D) or the trained hyperparameters, in case they were optimized.

SM5.1.2 Generalized linear model (GLM)

Generalized linear models are an extension of linear models that can incorporate non-Gaussian-distributed data (including discrete data). In MINT, the `glm_wrapper.m` function trains and test GLM models using lasso, ridge or elastic net regularization and either `lassoglm.m` (Statistics and Machine Learning Toolbox in MATLAB) or the `glmnet` toolbox [36]. This function allows for the training and testing of GLM models with optional regularization methods, including lasso, ridge, or elastic net. It provides flexibility in choosing regularization types and additional options for k-fold cross-validation. Depending on the option chosen in `reqOutputs`, it outputs as $\hat{\boldsymbol{R}}$ an array of cross-validated predicted labels for each trial, or cross-validated posterior probabilities of $\boldsymbol{s}$ given the neural data in each trial.

SM5.2 Interfacing information calculation with unsupervised dimensionality-reduction method

Unsupervised methods transform neural data into a lower dimensional output $\hat{\boldsymbol{R}}$ that still approximate the data well. Our routines take input data in the form of a list across all trials of neural responses $(\boldsymbol{r_1}, \dots, \boldsymbol{r_N}) \in \{\boldsymbol{R_1}, \dots, \boldsymbol{R_N}\}$, as well as the desired dimensionality of the reduced representation. It returns a list of reduced representation $\hat{\boldsymbol{r}}$ of $(\boldsymbol{r_1}, \dots, \boldsymbol{r_N})$. The reduced

neural representation data are then fed as neural data input to the information routines for information calculation. Unsupervised models implemented in MINT are describe below.

SM5.2.1 Principal Component Analysis (PCA)
Principal Component Analysis (PCA) is a dimensionality reduction technique that uses an orthogonal linear transformation to project the data onto a lower dimensional space with maximal variance (REF). MINT's function `pca_wrapper.m` outputs in each trial the coefficients of the data along the selected number of principal components.

SM5.2.2 Non-negative Matrix Factorization (NMF)
Non-negative Matrix Factorization (NMF) is another technique of dimensionality reduction that projects the data onto a lower-dimensional space that still describes the data well. Unlike PCA, it does not require different components to be orthogonal, but requires that the decomposition is performed with nonnegative coefficients and basis functions (which is recommended for reducing the dimensionality of inherently nonnegative data, such as spike counts). MINT's function `nmf_wrapper.m` outputs in each trial the non-negative coefficients of the data along the selected number of principal components.

SM6 Details of simulations

SM6.1 Simulations of neural populations information encoding
This section presents a detailed description of the simulation and the analysis of information encoding in neural populations presented in Fig. 2.

We simulated three scenarios which capture main ways in which correlations have been reported to influence population coding [18, 37, 38]. For each scenario we simulated correlated spike trains of a neural population of N = 20 neurons responding to two simulated stimuli (200 trials per stimulus, 10 simulation repetitions).

The strength of correlations between neurons was modulated by generating responses to each stimulus as the sum of an independent Poisson process (independent outcome for each neuron) and a shared Poisson process (same outcome across neurons), adjusting the pairwise Pearson noise correlation for each stimulus by varying the contribution of the shared and independent processes to the spike trains. Thus, the spike count of neuron i $(i = 1,2)$ was generated as:

$$r_i(s) = r_{i-individual}(s) + r_{shared}(s) \quad (6)$$

where $r_{i-individual}(s)$ and $r_{shared}(s)$ are the output of 3 independent Poisson processes for each stimulus, with mean count parameter indicated by the corresponding name.

The first scenario (Fig. 2A) was implemented with strong stimulus modulation of the correlation strength, resulting in information-enhancing noise correlations. The individual and

shared processes were created such that the resulting total firing rate of each of the neurons is constant across stimulus values, so only the firing correlation is informative about the stimulus. The parameters were $r_{i-individual}\ (s = 1) = 1\ sp/s$, $r_{shared}\ (s = 1) = 1\ sp/s$, $r_{i-individual}\ (s = 2) = 2\ sp/s$, $r_{shared}\ (s = 2) = 0\ sp/s$.

For the second scenario (Fig. 2B) we simulated information limiting noise correlation. Namely we simulated a population of neurons all with the same stimulus selectivity (lower spiking rate to the first stimulus and a higher spiking rate to the second stimulus and thus positive signal correlations) and with positive noise correlations that were only weakly stimulus dependent. The parameters were $r_{i-individual}\ (s = 1) = 0.8\ sp/s$, $r_{shared}\ (s = 1) = 0.2\ sp/s$, $r_{i-individual}\ (s = 2) = 1.9\ sp/s$, $r_{shared}\ (s = 2) = 0.1\ sp/s$.

A third scenario was used for the simulations of the bias in Fig. C panel B and is described next. We simulated a population of Poisson neurons whose mean rate was stimulus-selective similar to the previous stimulation (lower spiking rate to the first stimulus and a higher spiking rate to the second stimulus). The parameters were $r_{i-individual}\ (s = 1) = 1\ sp/s$, $r_{shared}\ (s = 1) = 0\ sp/s$, $r_{i-individual}\ (s = 2) = 2\ sp/s$, $r_{shared}\ (s = 2) = 0\ sp/s$. Since we did not add any shared process, the neurons were independent from each other at fixed stimulus and there was no noise correlation. This facilitate the numerical computation of the ground-truth values of information for this process, which were used in the study of the bias properties.

The so generated spike counts were binned into 5 bins, by leaving spike counts ≤ 4 untouched and setting to 4 all spike count values ≥ 5 (this was done by setting input options `opts` of `MI.m` the `binning_method` field `'userEdges'`, that allows binning the data with user-defined bin edges). All MI and PID values were corrected for the limited-sampling bias by using the shuffle-subtraction procedure implemented in the toolbox (averaged over 30 shuffles).

We used the `svm_wrapper.m` function of the toolbox to predict the stimulus based on the population activity by fitting a cross-validated Support Vector Machine (SVM) with 2 folds. Two distinct kernel functions were employed to fit the SVM to the data: linear and RBF. We performed hyperparameter optimization, tuning the parameters C for linear SVM and C and gamma for SMV RBF using 2 folds cross-validation. We used Bayesian optimization over a logarithmic scale ranging from 10^{-3} to 10^{3} and maximum number of iterations equals 30. To evaluate the role of correlations in information encoding we computed the Mutual Information of the predicted and the true stimuli. To eliminate noise correlations, pseudo-responses were generated by shuffling the simulated response conditionally on the stimulus value, so they have the same single cell properties as the original data but no noise correlations (h`Shuffle.m` function, `reqOutputs` defined as `'A_B'` and `'dim_shuffle'` set to `'Trials'`). We computed the Mutual Information using the `MI.m` function of the toolbox between the actual stimulus and the one predicted from all above-described decoders (linear and RBF kernel SVM for simulated response and pseudo-response) and compared them to gain insights into the

effects of noise correlation on the population information. The simulation was repeated n = 10 times and results were averaged across repeats.

SM6.2 Simulation of encoding and readout of information in pairs of neurons

This section presents a detailed description of the simulation and analysis of information encoding and readout from pairs of neurons presented in Fig. D.

We simulated a pair of neurons, independently encoding a binary stimulus S with values $s \in [-1,1]$ across 1000 trials. The single-trial firing rate of each neuron $i \in 1,2$ was determined by a Poisson process $r_i(S) \sim Poisson(\lambda_i(S))$, with the intensity parameter $\lambda_i(S)$ depending on the stimulus as $\lambda_i(S) = \lambda_0 + \Delta \cdot W_{enc,i} \cdot S$. λ_0 determined the mean firing rate of each neuron across trials, Δ the separation in the mean firing rates across the two stimuli, and $W_{enc,i}$ was the element i of the 2-dimensional encoding vector $\overline{W}_{enc}$ determining the tuning of each neuron to the stimulus. In our simulations we set $\lambda_0 = 4$, $\Delta = 1$ and $\overline{W}_{enc} = (1,1)$, so that both neurons had lower firing rates for $s = -1$ and higher firing rates for $s = 1$.

On each trial, we simulated a choice variable C by taking the dot product between the population firing rates and a decoding vector $\overline{W}_{dec}$, such that $C = \bar{r} \cdot \overline{W}_{dec}$ and binarized C using equi-populated binning. We obtained the decoding vector $\overline{W}_{dec}$ by applying the standard two-dimensional rotation matrix $R_2(\theta)$ to the encoding vector $\overline{W}_{enc}$ as follows $\overline{W}_{dec} = R_2(\theta) \cdot \overline{W}_{enc}$.

We used the `svm_wrapper.m` function of the toolbox to train a cross-validated (5 folds) linear SVM (hyperparameter $C = 1$) to decode the stimulus $\hat{S}$ and the choice $\hat{C}$ from the neural population activity. We computed stimulus, choice and intersection information as $I(S,\hat{S})$, $I(C,\hat{C})$ and $II(S,[\hat{S},\hat{C}],C)$ respectively, using the `MI.m` and the `II.m` functions of the toolbox (with no bias correction).

We simulated two different scenarios, one with a small ($\theta = 20°$) and one with a large ($\theta = 70°$) angle between the encoding and the decoding vectors. We computed the angle between the decision boundaries of the SVM trained to decode the stimulus ($\overline{W}_{\hat{S}}$) and the one to decode the choice ($\overline{W}_{\hat{C}}$) as

$$\hat{\theta} = \arccos\left(\frac{\overline{W}_{\hat{S}} \cdot \overline{W}_{\hat{C}}{}'}{||\overline{W}_{\hat{S}}|| \cdot ||\overline{W}_{\hat{C}}||}\right) \tag{7}$$

where the prime symbol ($'$) indicates the transpose operation, and $||\overline{W}||$ indicates the norm of vector $\overline{W}$. A total of 5 simulations were conducted for each scenario, information and angle values were averaged across simulations.

SM6.3 Simulation of aggregate activity signals in networks of interacting nodes

This section presents a detailed description of the simulation and analysis of aggregate signal activity in a network of interacting nodes (Fig. 4 and Fig. E).

The network implemented content-specific encoding and transmission of information across four nodes, X_1, X_2, X_3 and X_4 (N = 4) each of them divided into two subnodes $X_{N,1}$ and $X_{N,2}$ (M = 2). A total of 10 simulations were conducted, each including 200 trials with a duration of 30 ms. We simulated a stimulus that included two binary features S_1 and S_2 whose values were drawn independently in each trial. The value of $S_1(t)$ was set equal to the value of $s_1 \in [-1,1]$ while the value to $S_2(t)$ was set equal to the value of $s_2 \in [-1,1]$ within a defined stimulus-active time window. Outside this window, the value was set to zero.

Subnodes $X_{1,1}$ and $X_{4,1}$ received input regarding S_1, while subnode $X_{2,2}$ received input regarding S_2. The stimulus-active time window for $X_{1,1}$ and $X_{2,2}$ was defined as [3, 12] ms, while $X_{4,1}$ received the input with a delay Δt_2 of 12 ms (stimulus-active time window [15, 24] ms). In addition, all subnodes received stimulus-feature unrelated activity at any timepoint as zero-mean Gaussian noise $\mathcal{E}_{N,M}(t) = N(0, \sigma_{noise})$ with standard deviation $\sigma_{noise} = 0.5$.

To simulate the transfer of feature-related and unrelated activity from one subnode to another, a delay Δt_1 of 5 ms was defined. With that time delay, subnode $X_{1,1}$ transmitted its activity to $X_{2,1}$ and $X_{3,1}$, subnode $X_{2,2}$ to $X_{1,2}$ and $X_{3,2}$ transmitted its activity to $X_{4,2}$.
The aggregated activity of each node is defined as the summed activity of the two subnodes. The activity of the four nodes at each time point is defined as:

$$X_1(t) = X_{1,1}(t) + X_{1,2}(t) = \left(\alpha \cdot S_1(t) + \mathcal{E}_{1,1}(t)\right) + \left(X_{2,2}(t - \Delta t_1) + \mathcal{E}_{1,2}(t)\right) \quad (8)$$

$$X_2(t) = X_{2,1}(t) + X_{2,2}(t) = \left(X_{1,1}(t - \Delta t_1) + \mathcal{E}_{2,1}(t)\right) + \left(\alpha \cdot S_2(t) + \mathcal{E}_{2,2}(t)\right) \quad (9)$$

$$X_3(t) = X_{3,1}(t) + X_{3,2}(t) = \left(X_{1,1}(t - \Delta t_1) + \mathcal{E}_{3,1}(t)\right) + \left(\mathcal{E}_{3,2}(t)\right) \quad (10)$$

$$X_4(t) = X_{4,1}(t) + X_{4,2}(t) = \left(\alpha \cdot S_1(t - \Delta t_2) + \mathcal{E}_{4,1}(t)\right) + \left(X_{3,2}(t - \Delta t_1) + \mathcal{E}_{4,2}(t)\right) \quad (11)$$

For all subsequent analysis we binned the activity of each node into $R = 3$ equi-populated bins and we corrected for the limited-sampling bias of information with the QE procedure implemented in the toolbox.

First, we computed the Mutual Information between each node and the stimulus features at all time points (see Fig. E panel B), using the `MI.m` function of the toolbox.

Consistent with the implemented stimulus input and information transfer, X_1 exhibited information about S_1 from 3 to 12 ms, while X_2 and X_3 displayed a 5 ms delayed stimulus-feature informative window. The simulated activity of X_4 contained information regarding S_1 from 15 to 24 ms, consistent with the implemented input delay of 12 ms compared to X_1. Information about S_2 was only present in X_1 ([8, 15] ms) and X_2 ([3, 12] ms), in line with the implemented input of S_2 and delayed transfer from X_2 to X_1.

To gain insight into the information transfer within the network we computed the transfer entropy between all pairs of simulated nodes, using the `TE.m` function provided by the toolbox. Based on the established ground truth, the temporal parameters to compute transfer entropy were defined as t = 12 ms and Δt = 5 ms. Consistent with the implemented network interactions, we found significant transmission of information from X_1 to X_2 and X_3, from X_2 to X_1 and from X_3 to X_4 (see heatmap in Fig. E panel A).

When analyzing real neural data, the optimal time and delay parameters are typically not known. Therefore, we demonstrate in the next step how one can assess these parameters by computing the time-delay maps of information transmission between nodes X_1 and X_2. To compute the content of information flow, we measured FIT with the `FIT.m` function implemented in the toolbox. To obtain the temporal profile of content-specific information transmission and to reconstruct the delay of information transmission, we first computed FIT at each time step of the simulation with all possible delays for $X_1 \rightarrow X_2$ related to S_1, $X_1 \rightarrow X_2$ related to S_2 and for $X_2 \rightarrow X_1$ related to S_2 (see Fig. E panel C). Consistent with the implemented ground truth, significant transfer of information was found with a delay of 5 ms from X_1 to X_2 related to S_1 and from X_2 to X_1 related to S_2.

By computing FIT for all pairs of nodes (t = 12 ms, Δt = 5 ms), we found significant information transfer related to S_1 from X_1 to X_2 and X_3 and significant information transfer related to S_2 from X_2 to X_1 (see Fig. 4D and Fig. E panels A and C).

To test for significance in the information theoretic quantities averaged across the n = 10 simulations, we used non-parametric permutation tests. For each simulation, we first conducted two different shuffling procedures 100 times and recomputed TE and FIT from the shuffled data (t = 12 ms, Δt = 5 ms). First, we conditionally shuffled the sender activity at fixed value of the stimulus to preserve stimulus induced covariations between the sender and the receiver and destroy single-trial correlations contributed by real communication. Second, we shuffled S for FIT to break any relationship between the stimulus and variables X and Y, and we shuffled X for the TE analysis to break any relationship between X and Y. We then took the pairwise maximum between the information values obtained from the two shuffling procedures to obtain a single, conservative null distribution [23]. Using the `create_nullDist_groupLevel.m` function of the toolbox we generated 500 samples of the null distribution of the permuted average across simulations, and estimated TE and FIT p-values empirically. To estimate the significance of FIT in the time-delay domain (Fig. E panel C), we implemented the same procedure outlined above computing FIT at each time step of the simulation with all possible delays. We then used cluster permutation (with the `pool` option set to 0) setting both `clusterPercentilThreshold` and `significanceThreshold` to the 99th percentile to individuate significant FIT clusters.

SM6.4 Simulation study of the limited sampling bias with various estimators and bias correction methods

This section presents a detailed description of the results of simulations of the activity of populations of neurons to illustrate and study the limited-sampling bias on the calculation of mutual information (Fig. C).

We first illustrated (Fig. C, Panel A, 'Sketch of bias for a zero-information process') the meaning and origin of the limited-sampling bias using a simple simulated example of two uninformative neurons. These neurons respond on each trial with a uniform distribution of spike counts ranging from 1 to 4, regardless of which stimulus values were presented. The colorplots in Fig. C, left side of Panel A show the joint and marginal probability distributions sampled from 50 simulated trials per stimulus in a single simulation. Despite the underlying distributions are completely uniform with respect to the stimulus or response value, the stimulus-specific probability distributions of the joint neural-pair or marginal single-neuron responses empirically sampled from a limited number of trials differ across stimuli because of the limited sampling. A naïve interpretation of this difference would make one conclude that since the response distribution changes for each stimulus, the neural response carries some information about the stimulus. When computing many (50000) repeated simulations of this uninformative process with 50 trials per stimulus each, the distribution of plugin information values computed with the direct-method (Fig. C, right side of panel A) did not have a mean equal to ground-truth information value of zero, but a different, positive mean. The bias is the difference between the ground-truth value of information and the one obtained on average across different random instantiations of a dataset with a given limited number of trials. In this case, the bias is 0.022 bits for the single neuron information and 0.12 bits for the joint information in the activity of the neuron pair. The reason why the limited-sampling bias is larger for the neuron pair than the single neuron can be appreciated by considering the example single instantiation of the empirical probability Fig. C, Panel A, left. The random fluctuations that generate spurious information are larger for the joint probability (the few trials are spread over a larger set of possible responses $R = 16$) than for the marginal probabilities (the few trials are spread over a smaller set of responses $R = 4$). As a result of these spurious differences between stimulus-specific response distributions, the values of information computed from individual simulations with the limited number of trials (right panel) would not be distributed around the true value of zero bits, but around a spurious non-zero value (the bias). Since the fluctuations are larger in the joint probability estimation than for the marginal distributions, the bias values would be bigger in this case in comparison to the information computed from the single neuron information distributions.

To further illustrate how the limited-sampling bias scales with the number of available trials, in Fig. C Panel B ('Bias for a stimulus-informative Poisson process') we studied the performance of the estimation algorithms considering Poisson spike counts of populations of neurons ranging from $N=1$ to $N=4$, and carrying information about a binary stimulus. We used Poisson neurons because for this process we could evaluate numerically with high precision the ground-truth value of the information carried by the population. This ground truth value can be computed by inserting the analytical probabilities of the Poisson process into the Shannon formula and stopping the sum over responses at some value of spike counts much higher than the average mean spike count of the Poisson process (probabilities converge to zero

for such very high spike counts and thus the sum over responses can be stopped at some point with negligible loss of numerical accuracy). To simulate what we could do in real data, we binned spike counts of each neuron into R =5 bins (0, 1, 2, 3, >3 spike counts). We then used the direct-method calculation without bias corrections (plugin) and with two bias-correction methods (QE, shuffle-subtracted). For comparison, we also computed the information estimated through a method popular for the analysis of real-valued data (KSG), which is also available in MINT. We also plotted the ground-truth value of information computed from the analytical form of the probability distributions of the Poisson spike counts after binning the data. The QE bias correction was done with 10 repetitions (`xtrp`=10). The shuffle-subtraction was done with 30 shuffles (`shuff`=30).

As documented in previous studies [27], the limited-sampling bias of the bias-uncorrected, plugin, estimator is very high for low numbers of trials and then smoothly decreases with the number of trials. The bias also grows very rapidly with the population size. Application of QE or shuffle-subtraction bias correction to the plugin estimators provides an effective elimination of the limited-sampling bias for number of trials that are realistic with a neuroscience experiment (~100) for N=1,2 but not for N>2. The KSG method, not being designed for discrete spike count processes, does not converge to the ground-truth information value even for large trial numbers, with a data-processing bias that grows with N.

The above simulations indicate a distinction between two types of bias. The first is the (usually upward) limited-sampling bias, which is the difference between the value of information obtained with the considered estimation method (e.g. response binning, KSG, etc.) when considering a finite number of trials vs when considering an infinite number of trials. The second is the data-processing bias, which reflects the possible inability of the estimation method to process or represent the probability distributions of the data correctly. The data-processing bias can be computed as the difference between the ground truth value of information and the one obtained with the considered estimation method if the data were infinite. For example, when discretizing neural responses into a more limited number of possible discrete responses than the ones that could actually happen (for example, binarizing as all/none the output of a Poisson count), the data estimation bias would be negative.

When using the direct-method discrete estimators with binned responses, we found that the direct method had a negligible data-processing bias. The direct-method discrete information estimator without bias correction is however upward biased for large numbers of trials. The bias corrections algorithms QE and shuffle-subtract worked well and made the estimate convergence close to the ground truth value even for a relatively small number of trials. In contrast, the KSG has a relatively small limited sampling bias but at a cost of a large downward data-processing bias, which reflects the fact that its assumption does not suit discrete data as well as real valued data. We thus would not use the KSG for computing information from neural spike counts.

We next investigated (Fig. C, panels C and D) the interplay between data dimensionality reduction and limited sampling bias. We simulated the two scenarios of correlated neural population activity that were used in Figure 2A,B and that are detailed in Section SM6.1. We then implemented different dimensionality reduction algorithms to reduce the 20-dimensional neural data to 1 dimension. The activity of the 20 neurons was reduced to one dimension using either `svm_wrapper.m` for linear or RBF SVM or `pca_wrapper.m` for PCA. All dimensionality reduction methods were crossvalidated using 2 folds.

We first considered a case in which correlations between activity of neurons are present but are not much informative and the information about the stimulus is by and large specified by differences in average spike counts of individual neurons across stimuli. This case is equal to the one presented in Fig 2B and the results of the simulations are reported in Fig C, Panel C ('Dimensionality reduction with information in single neurons spike counts'). The information estimates from the reduced 1-dimensional representation show little dependence on the sample size and are almost identical between corrected and uncorrected (plugin) estimates, suggesting that the dimensionality reduction has little bias and that calculation of information from a population of 20 neurons can be performed robustly with limited data sizes when using dimensionality reduction. All dimensionality reduction methods perform similarly, because it is very easy for all methods to find the dimension in neural activity space with maximal information (which is simply the vector of the averaged differences in spike counts between the two stimuli) in a case in which the information is encoded in major differences in spike counts across stimuli.

We finally considered a case in which spike counts of individual cells did not carry information (average spike counts were constant across stimuli) but all information was encoded in the stimulus variations of the strength of correlations between activity of different neurons. This case is equal to the one presented in Fig 2A and the results of the simulations are reported in Fig C, Panel D ('Dimensionality reduction with information in correlations'). In this case, and also shown in Fig 2A, the information can be recovered only with a complex non-linear decoder (RBF SVM) for any considered number of trials. Limited sampling bias appears small but the increase of RBF SVM suggests that more complex supervised decoders benefit from more training data to reduce the data processing bias.

In conclusion, for tens of neurons the limited sampling bias can be well controlled for using dimensionality reduction methods. Most algorithms extract similar amounts of information even with little training data when information is encoded in very easily detectable features of neural activity (e.g. clear spike count separations) while more sophisticated algorithms (e.g. non-linear supervised decoders) and more training data are needed when information is hidden only in more subtle features of neural activity such as the correlations between their activity.

SM7 Supplemental details and supplemental results of analyses of real neural data

SM7.1 EEG analysis methods

We analyzed a publicly available EEG dataset [39] (available at https://datadryad.org/stash/dataset/doi:10.5061/dryad.8m2g3). Full details are reported in the original publication. Here we summarize them briefly. The EEG data were recorded while participants (n=16) performed a face detection task. Participants were presented with an image hidden behind a bubble mask that was randomly generated in each trial. The presented image was an image of a face in half of the trials and a random texture in the other half of the trials. Participants were instructed to report whether a face was present or not. In our analyses, we only considered trials where the face was correctly detected by the participants (approximately 1000 trials per subject). Following the recommendations of the original publications analyzing these data [99,100], we excluded one participant from the analysis. All analyses in our paper are based on the n=15 selected participants. EEGs were recorded by fitting participants with a Biosemi head cap comprising 128 EEG electrodes. EEG data were re-referenced offline to an average reference, band-pass filtered between 1 Hz and 30 Hz using a fourth order Butterworth filter, down-sampled to 500 Hz sampling rate and baseline corrected using the average activity between 300 ms pre-stimulus and stimulus presentation. ICA was performed to reduce blink and eye- movement artifacts (see [39, 40]).

For the analyses of TE and FIT, we selected the EEG electrodes in the left and the right Occipito-Temporal regions that had the highest Mutual Information about the visibility of the contra-lateral eye, exactly as done in previous papers [23, 40]. We computed the first derivatives of the EEG signal for both Occipito-Temporal sensors and used both their absolute values and first derivatives to compute the information quantities, for consistency with analyses performed in previous studies [23, 40]. As stimulus feature for the computation of Mutual Information and FIT, we used the visibility of an eye (defined as the fraction of pixels within the eye region that were not hidden by the bubble mask). Both neural and stimulus features were discretized using 2 equi-populated bins. We computed the information quantities for all combinations of directionality of flow across hemispheres (left to right, right to left) and eye identity (left or right eye). As done in previous papers REF to compute a single TE and FIT value for each participant we selected a rectangular region in the time-delay domain centered around the contra-lateral FIT peaks (time ranging from 140 ms to 240 ms peri-stimulus presentation, delay ranging from 20 ms to 90 ms; same for both eyes, as they were significant in very similar time-delay regions). We computed the average over delays and then picked the maximum over time within this region. We used the same procedure described in SM6.3 to compute the significance of the across-participants averaged FIT and TE (Fig. 4G-H), generating 500 null samples from 10 shuffles within each participant.

Files that reproduce the analysis of these data are found in https://github.com/panzerilab/MINT_figures, subfolder 'Figure4'.

SM7.2 Analysis of CA1 data

We reanalyzed a previously published dataset [41] in which the activity of several tens to a few hundreds of neurons was recorded simultaneously using in-vivo two-photon calcium imaging from CA1 neurons in head-fixed transgenic mice during virtual reality navigation of a linear track. This dataset is provided as Supplemental Information file 'S2_CA1_data.mat'.

We analyzed neurons recorded from $n_{FOV} = 11$ Fields of View (FOV) from $n_A = 7$ animals. For consistency with the previous study reporting the original data [27], the spatial position of the linear track was computed by binning the space along the track into $S = 12$ equi-populated bins. Also, the neural activity r_i of each neuron was quantified by binning the calcium traces into $R = 2$ equi-populated bins (only raw calcium traces and not deconvolved signals were available from [41]. For the PID analysis, we used all individual neurons present in the dataset leading to $n_{pairs} = 36158$ pairs of simultaneously recorded neurons used for the pairwise direct information analyses and to $n_{sessions} = 11$ sessions for the population vector analyses. The neural responses dimensionality was reduced using linear and nonlinear (RBF) SVM to predict the position categories using 5-fold cross-validation and hyperparameter optimization (2-fold cross-validation, Bayesian optimization over a logarithmic scale ranging from 10^{-3} to 10^3 for C and gamma and maximum number of iterations equals 30) and later the predicted labels were used jointly to replace the full neural response.

Files that reproduce the analysis of these data, as well as the neural data themselves, are found in https://github.com/panzerilab/MINT_figures, subfolder ‘Figure2’.

SM7.3 Analysis of A1 data

We reanalyzed a previously published dataset [42] in which the activity of several neurons was recorded simultaneously using in vivo two photon calcium imaging from A1 L2/3 neurons in head-fixed transgenic mice during a pure-tone discrimination task. Data are publicly available at https://doi.org/10.13016/m2yt-mfxk .

The experimental task was structured as follows. After a pre-stimulus interval of 1 s, head-fixed mice were exposed to either a low-frequency (7 or 9.9 kHz) or a high-frequency (14 or 19.8 kHz) tone for a period of 1 s. Mice were trained to report their perception of the sound stimulus by their behavioral choice, which consisted of licking a waterspout in the post-stimulus interval (0.5–3 s from stimulus onset) after hearing a low-frequency tone (target tones) and holding still after hearing high-frequency tones (non-target tones). Two-photon calcium imaging was used to acquire the calcium fluorescence signals from individual A1 L2/3 neurons during the task with an imaging frame rate of 30 Hz. We pre-processed these data as follows to match the pe-processing used by the authors in the original publication. We smoothed the raw calcium fluorescence traces using a zero-phase (MATLAB `filtfilt.m` function) order-2 low-pass Butterworth filter (`butter.m` function in MATLAB) with normalized cutoff frequency of f/(fs/2), where f=2Hz is the low-pass cutoff frequency, and fs=30 Hz is the sampling frequency of the calcium imaging data. As in the original publication [42] the resulting traces were deconvolved with a first-order autoregressive model.

We analyzed neurons recorded from n= 12 Fields of View (FOV) from n = 12 animals. For consistency with the previously published work, we only considered the 20 individual neurons in each session with the shortest-latency intersection information peak, as described in [42]. This led to selecting n = 2280 pairs of simultaneously recorded neurons used for the pairwise

direct information analysis and n=12 sessions for the population analyses. For the information analysis of these data, we identified for each neuron the imaging time frame within the trial of maximal intersection information exactly as in the original publication [42].

We then considered for each neuron a time frame of n = 10 imaging frames (corresponding to a window of 333 ms) around the peak intersection information time frame (we call this the peak time window for the neuron). Then we discretized activity for each neuron into R = 3 bins according to whether it was detected 0, 1 or > 1 spikes in the peak time window (this was done by setting in input options `opts` of `MI.m` the `binning_method` field `'userEdges'`, that allows binning the data with user-defined bin edges). The stimulus set used for the stimulus encoding analysis was binary, dividing the presented sound tones into the low- and high-frequency categories. The choice set used for the intersection information analysis was also binary (lick vs no lick). For Figure 3, we used the `svm_wrapper.m` function of the toolbox to train a cross-validated (2 folds) RBF SVM (hyperparameter $C = 1$) to decode the stimulus $\hat{S}$ and the choice $\hat{C}$ from the neural population activity. We computed stimulus and intersection information as Mutual Information $I(S, \hat{S})$ between presented and decoded stimulus and II as the Intersection Information $II(S, [\hat{S}, \hat{C}], C)$ between the presented stimuli, the mouse choices and the stimulus and choice decoded from neural activity using the `MI.m` and the `II.m` functions of the toolbox. The bias was corrected by applying the shuffle subtraction procedure setting the `shuff` field of the `opts` structure to 30. Figure B illustrates the pipeline of dimensionality reduction and information measurement used to generate Fig. 3. The fitting was performed using a second-order polynomial on the logarithm of the population size.

Files that reproduce the analysis of these data are found in https://github.com/panzerilab/MINT_figures, subfolders 'Figure2' and 'Figure3'.

Supplementary Figures and Figure Captions:

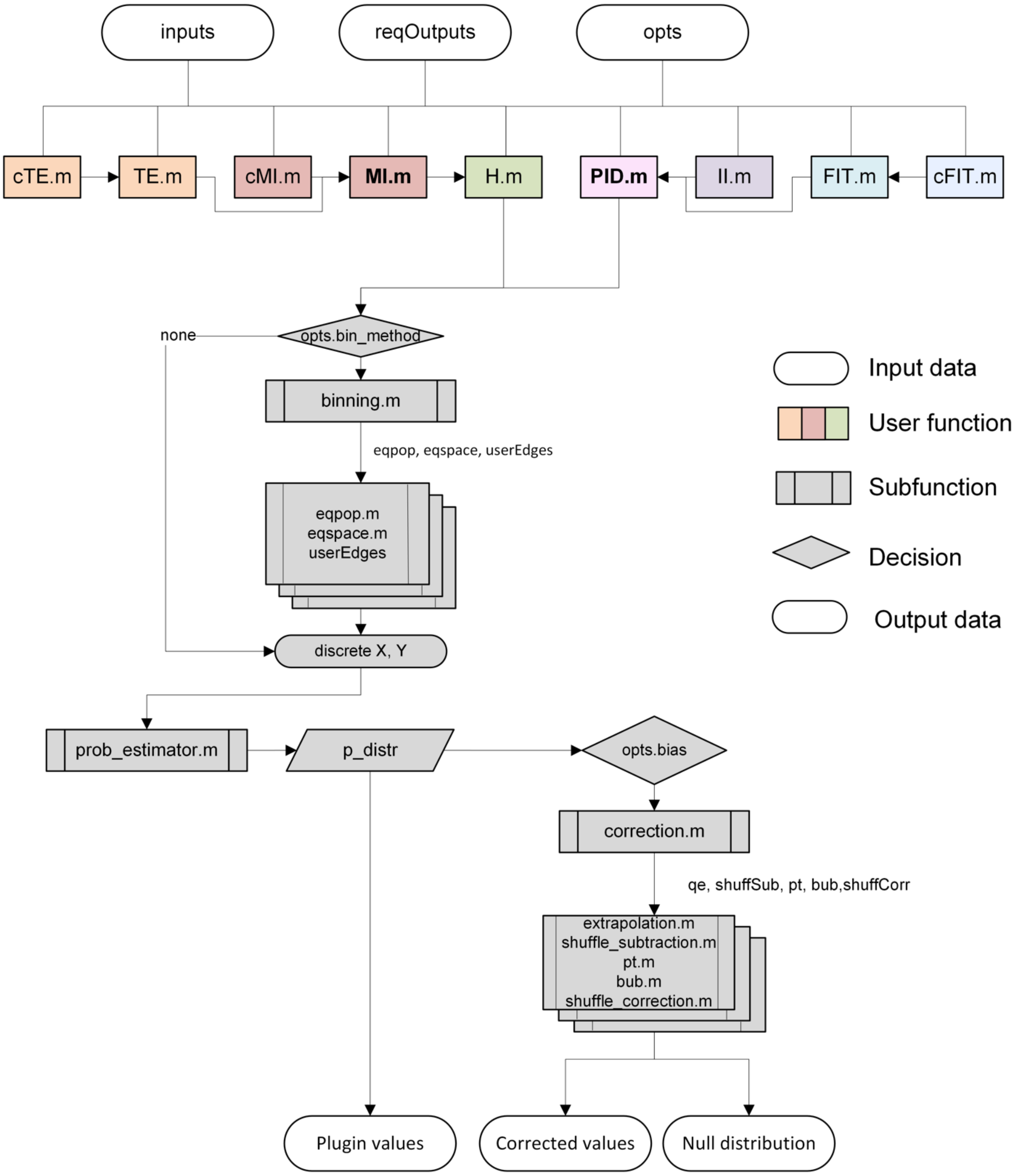

Figure A. *Flowchart of the MINT toolbox*. The flowchart illustrates the structure and workflow of the MI module of the Toolbox, highlighting the steps involved in computing information values.

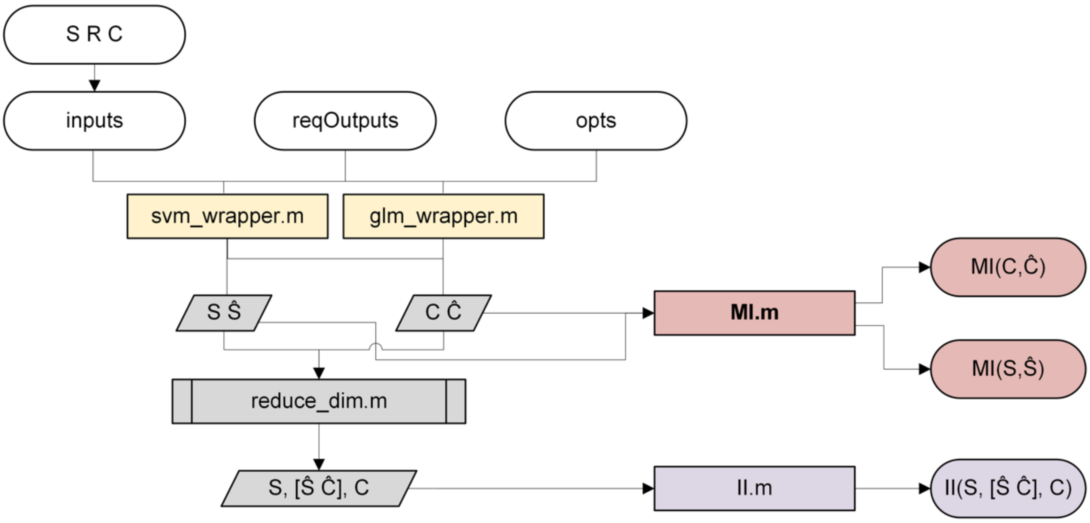

Figure B. *Example flowchart of the pipeline module used with the MI and II functions.* This is an example pipeline using the dimensionality reduction wrappers in the toolbox with information-theoretic functions. One could also use the unsupervised wrappers (PCA or NMF) to do the dimensionality reduction or any other of the information functions (PID, TE, etc.).

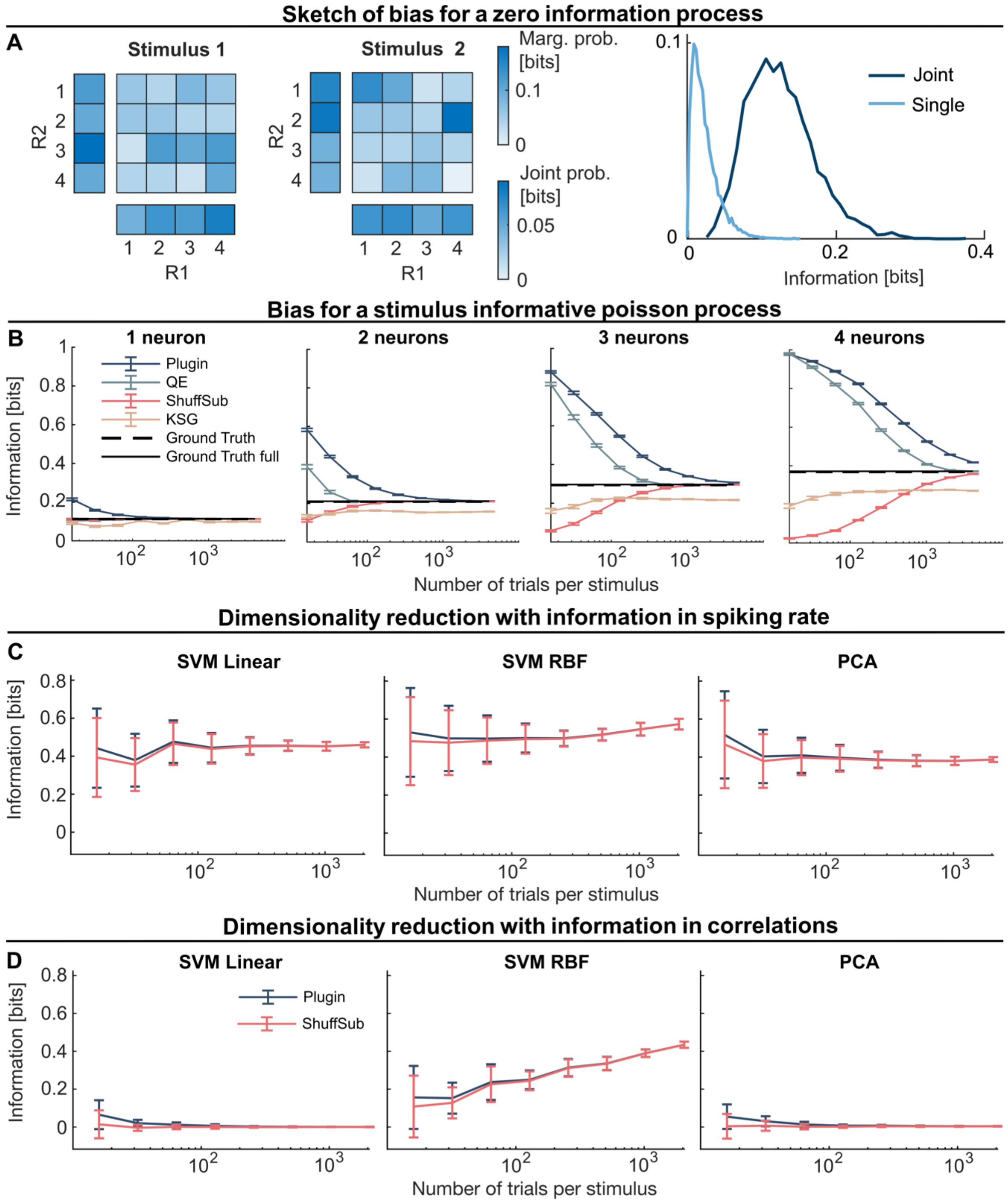

Figure C: *Limited-sampling bias of the direct method explodes with neural response dimensionality, but information can still be computed robustly with dimensionality reduction techniques.*

A: Schematic illustration of the limited-sampling bias problem. Two uninformative neurons, responding on each trial with a uniform distribution of spike counts ranging from 1 to 4, regardless of which 2 stimulus values were presented. The empirical response probability heatmaps sampled from 50 trials per stimulus are shown in the left and middle columns (responses to stimuli 1 and 2, respectively). Side heatmaps indicate the marginal probability values. Because of limited sampling, the neural response probabilities look different across

stimuli (even if they are not), and much more so for the joint probability than for the marginal response probabilities. Right: distribution (over 5,000 simulations) of the plugin information values obtained with 50 trials per stimulus. Although the information should be zero, it is > 0 because of the random sampling variations illustrated in panel A, left. The bias is the (non-zero) average value of this distribution, as the true asymptotic value should be zero. The bias is larger for the joint information (when neural activity is 2-D) than for the single neuron information (when neural activity is 1-D).

B: Mean ± SEM over 25 simulations, each performed with the number of trials per stimulus shown on the x axis, of Mutual Information $MI(R;S)$ between a binary stimulus S and a neural response $\boldsymbol{R}$ made of N Poisson uncorrelated neurons, with N varying from N=1 to N=4 from left to right. (see SI Section SM6.4). Spike counts of each neuron are discretized into R =5 bins (0, 1, 2, 3, >3 spike counts), and then information is computed with four different estimators (plugin, QE, shuffle-subtracted, KSG). We also plot the ground-truth value of information computed from the analytical form of the probability distributions of the Poisson spike counts either unbinned (full line, representing the exact value of information in the simulated process) or binned as described above (dashed line). The QE bias correction was done with 10 repetitions (`xtrp`=10). The shuffle-subtraction was done with 30 shuffles (`shuff`= 30). Application of QE or shuffle-subtraction bias correction to the plugin estimators provides an effective elimination of the limited-sampling bias for realistic number of trials <100) for N=1,2 but not for N>2. The KSG method does not converge to the ground-truth information value even for large trial numbers, with a data-processing bias that grows with N.

C: Mean ± SEM over 25 simulations, each performed with the number of trials per stimulus shown on the x axis, of Mutual Information $MI(R;S)$ between a binary stimulus S and a neural response $\boldsymbol{R}$ made of the spike counts of *N=20* correlated neurons, simulated as in Fig. 2B (see Section SM6.1). In this simulation, information is carried by design by the difference across stimuli in spike counts of individual neurons. The activity of the 20 neurons is reduced to one dimension using the `svm_wrapper.m` cross-validated with 2 folds when using the linear or RBF SVM or the `pca_wrapper.m` when considering PCA. The averaged information values do not depend much on the sample size and are almost identical between corrected and uncorrected (plugin) estimates, suggesting that the dimensionality reduction has little bias and that calculation of information from a population of 20 neurons can be performed robustly with limited data sizes when using dimensionality reduction.

D: Same as panel C, but for a population of N=20 neurons carrying information only by correlations without spike count modulations, simulated as in Fig. 2A. Here the information can be recovered only with a complex non-linear decoder (RBF SVM) for any considered number of trials. Limited sampling bias appears small but the increase of RBF SVM suggests that more complex supervised decoders benefit from more training data to reduce the data processing bias.

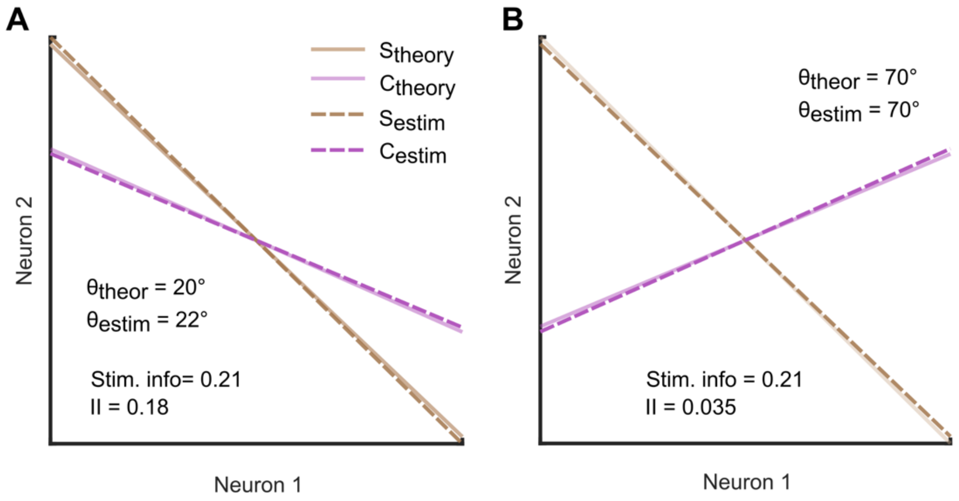

Figure D: *Role of stim-choice angle in the intersection information.*
Real and decoded stimulus and choice boundaries for a simulated population of 2 neurons with a small (A) and a large (B) angle between the stimulus and choice boundaries. The plot are made in the 2-D space of the firing rate of the two simulated neurons. This plot shows that MINT can reconstruct precisely the true neural activity axes that generate choices and stimulus coding. The figure also illustrates that, while stimulus information is the same for the two scenarios, the intersection information (II) is much larger for the case when stimulus and choice boundaries are well aligned. Simulations performed as detailed in Supplemental Material Section SM6.2

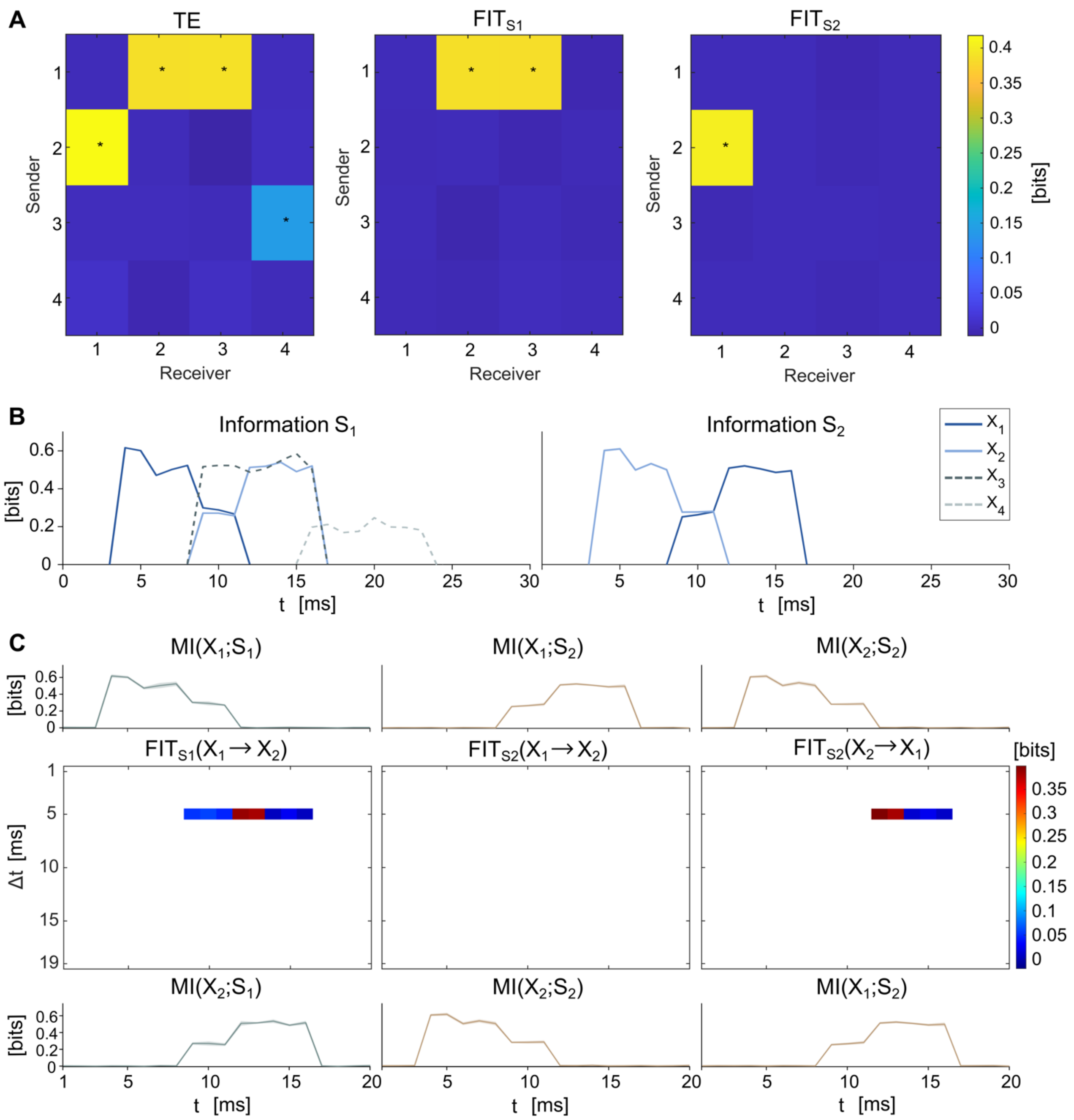

Figure E. TE and FIT heatmap, temporal profiles of stimulus-feature information and time delay stimulus-feature FIT maps for X_1 and X_2. **A:** TE heatmap for all nodes (left), FIT about S_1 for all nodes (middle), FIT about S_2 for all nodes (right) for timepoint t = 12 ms with a delay of 5 ms. Significant values (p<0.01) are marked with *. **B:** Mutual Information timecourses between the nodes X and stimulus feature S_1 and S_2. **C:** Analysis on specific node pairs: X_1 to X_2 about S_1 (left), X_1 to X_2 about S_2 (middle) and X_2 to X_1 about S_1 (right). **Top.** Mutual Information timecourse between the sender neural nodes and stimulus feature. **Middle.** FIT values across post-stimulus time t and delay time Δt from sender to receiver nodes about the stimulus feature. Only the time region with significant (p<0.01) stimulus information, according to a cluster permutation test with 500 null distribution samples, is plotted. **Bottom.**

Mutual Information between receiver and stimulus feature. Plots show the mean across n= 10 simulations.

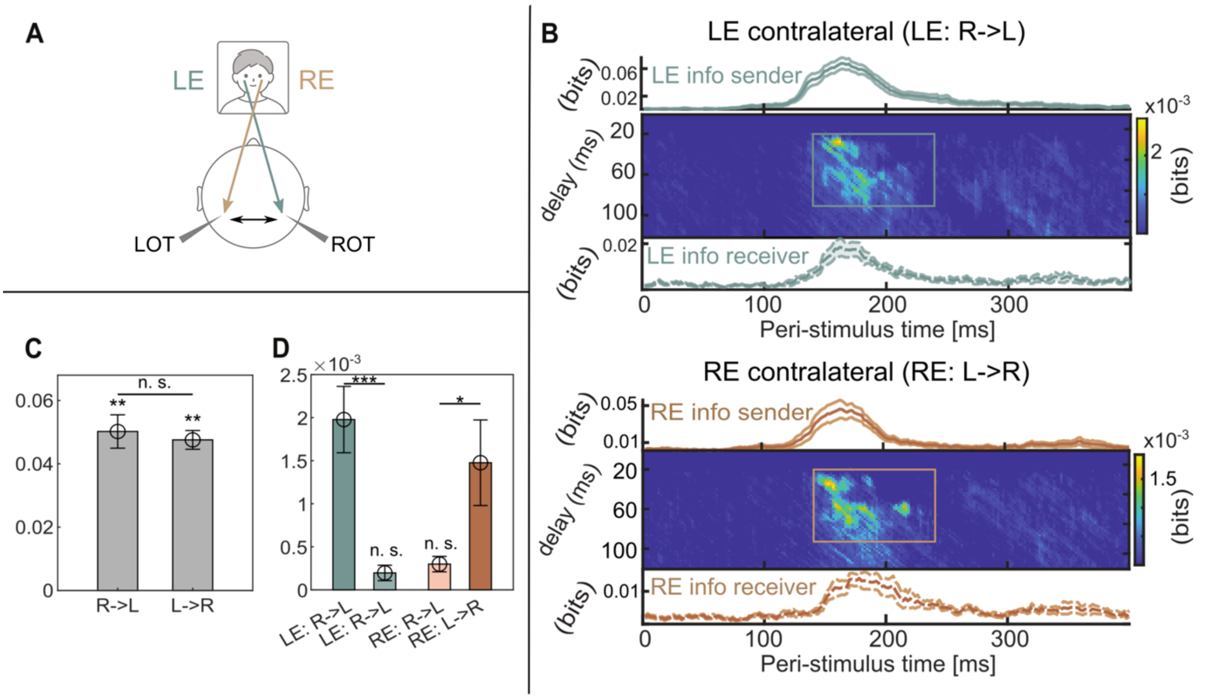

Figure F. A: Schematic of the putative information flow. LOT (ROT) denote Left (Right) occipito-temporal regions. LE (RE) denotes the Left (Right) Eye visibility feature. **B:** FIT values computed across post-stimulus time *t* and delay time from sender region to receiver region about the stimulus feature. The region of the time-delay maps used to calculate the final FIT values for Fig. 4H are delimited in both heatmaps. **Top.** Mutual Information (lines) carried by the EEG in each region, and FIT (image plot) about LE contra-lateral transfer. **Bottom.** Same as Top for RE **C:** Transfer Entropy between ROT and LOT in both possible directions. **D:** FIT values computed for each stimulus (LE or RE) and direction. In this plot ROT and LOT are written as R and L, respectively. Across all panels, dots and image plots show averages. Errorbars plot SEM across participants (n=15). Symbols *, **, *** denote two-tailed p<0.05, p<0.01, p<0.001 respectively, computed with paired t-tests. Human face sketch is modified from svgrepo.com/svg/493087/men-in-their-20s-and-30s-face, and head sketch is modified from doi.org/10.5281/zenodo.3926093. All resources are under license CC BY 4.0 (creativecommons.org/licenses/by/4.0).

Reference

1. Neri M, Vinchhi D, Ferreyra C, Robiglio T, Ates O, Ontivero-Ortega M, et al. HOI: A Python toolbox for high-performance estimation of Higher-Order Interactions from multivariate data. Journal of Open Source Software. 2024;9:7360. doi: https://doi.org/10.21105/joss.07360.
2. Combrisson E, Allegra M, Basanisi R, Ince RAA, Giordano BL, Bastin J, et al. Group-level inference of information-based measures for the analyses of cognitive brain networks

from neurophysiological data. NeuroImage. 2022;258:119347. doi: 10.1016/j.neuroimage.2022.119347.
3. Climer JR, Dombeck DA. Information Theoretic Approaches to Deciphering the Neural Code with Functional Fluorescence Imaging. eNeuro. 2021;8(5):ENEURO.0266-21.2021. doi: 10.1523/ENEURO.0266-21.2021. PubMed PMID: 34433574.
4. Candadai M, Izquierdo EJ. infotheory: A C++/Python package for multivariate information theoretic analysis. Journal of Open Source Software. 2020;57:1609. doi: 10.21105/joss.01609.
5. Makkeh A, Theis DO, Vicente R. BROJA-2PID: A Robust Estimator for Bivariate Partial Information Decomposition. Entropy. 2018;20(4). doi: 10.3390/e20040271.
6. Timme NM, Lapish C. A tutorial for information theory in neuroscience. eneuro. 2018;5(3).
7. James RG, Ellison CJ, Crutchfield JP. dit: a Python package for discrete information theory. Journal of Open Source Software. 2018;3:738. doi: 10.21105/joss.00738.
8. Ince RA, Giordano BL, Kayser C, Rousselet GA, Gross J, Schyns PG. A statistical framework for neuroimaging data analysis based on mutual information estimated via a gaussian copula. Hum Brain Mapp. 2017;38(3):1541-73. Epub 20161117. doi: 10.1002/hbm.23471. PubMed PMID: 27860095.
9. Moore DG, Valentini G, Walker SI, Levin M, editors. Inform: A toolkit for information-theoretic analysis of complex systems. 2017 IEEE Symposium Series on Computational Intelligence (SSCI); 2017.
10. Lizier JT. JIDT: An Information-Theoretic Toolkit for Studying the Dynamics of Complex Systems. Frontiers in Robotics and AI. 2014;1. doi: 10.3389/frobt.2014.00011.
11. Montalto A, Faes L, Marinazzo D. MuTE: A MATLAB Toolbox to Compare Established and Novel Estimators of the Multivariate Transfer Entropy. PLOS ONE. 2014;9(10):e109462. doi: 10.1371/journal.pone.0109462.
12. Szabó Z. Information theoretical estimators toolbox. The Journal of Machine Learning Research. 2014;15(1):283-7.
13. Lindner M, Vicente R, Priesemann V, Wibral M. TRENTOOL: A Matlab open source toolbox to analyse information flow in time series data with transfer entropy. BMC Neuroscience. 2011;12(1):119. doi: 10.1186/1471-2202-12-119.
14. Ito S, Hansen ME, Heiland R, Lumsdaine A, Litke AM, Beggs JM. Extending Transfer Entropy Improves Identification of Effective Connectivity in a Spiking Cortical Network Model. PLOS ONE. 2011;6(11):e27431. doi: 10.1371/journal.pone.0027431.
15. Magri C, Whittingstall K, Singh V, Logothetis NK, Panzeri S. A toolbox for the fast information analysis of multiple-site LFP, EEG and spike train recordings. BMC Neuroscience. 2009;10:81. doi: 10.1186/1471-2202-10-81.
16. Ince R, Petersen R, Swan D, Panzeri S. Python for information theoretic analysis of neural data. Frontiers in Neuroinformatics. 2009;3. doi: 10.3389/neuro.11.004.2009.
17. Goldberg DH, Victor JD, Gardner EP, Gardner D. Spike Train Analysis Toolkit: Enabling Wider Application of Information-Theoretic Techniques to Neurophysiology. Neuroinformatics. 2009;7(3):165-78. doi: 10.1007/s12021-009-9049-y.
18. Pola G, Thiele A, Hoffmann K, Panzeri S. An exact method to quantify the information transmitted by different mechanisms of correlational coding. Network: Computation in Neural Systems. 2003;14(1):35-60.
19. Chechik G, Globerson A, Anderson M, Young E, Nelken I, Tishby Ni. Group Redundancy Measures Reveal Redundancy Reduction in the Auditory Pathway. Advances in Neural Information Processing Systems. 2001;14:1.

20. Vicente R, Wibral M, Lindner M, Pipa G. Transfer entropy—a model-free measure of effective connectivity for the neurosciences. Journal of computational neuroscience. 2011;30(1):45-67.
21. Besserve M, Lowe SC, Logothetis NK, Scholkopf B, Panzeri S. Shifts of Gamma Phase across Primary Visual Cortical Sites Reflect Dynamic Stimulus-Modulated Information Transfer. PLoS Biol. 2015;13(9):e1002257. doi: 10.1371/journal.pbio.1002257. PubMed PMID: 26394205.
22. Wibral M, Pampu N, Priesemann V, Siebenhühner F, Seiwert H, Lindner M, et al. Measuring Information-Transfer Delays. PLOS ONE. 2013;8(2):e55809. doi: 10.1371/journal.pone.0055809.
23. Celotto M BJ, Tlaie A, De Feo V, Toso A, Lemke SM, Chicharro D, Nili H, Bieler M, Hanganu-Opatz IL, Donner TH, Brovelli A, Panzeri S An information-theoretic quantification of the content of communication between brain regions. Advances in Neural Information Processing Systems (NeurIPS). 2023;36:64213–65.
24. Runyan CA, Piasini E, Panzeri S, Harvey CD. Distinct timescales of population coding across cortex. Nature. 2017;548(7665):92-6.
25. Panzeri S, Harvey CD, Piasini E, Latham PE, Fellin T. Cracking the neural code for sensory perception by combining statistics, intervention, and behavior. Neuron. 2017;93(3):491-507.
26. Pica G, Piasini E, Safaai H, Runyan C, Harvey C, Diamond M, et al. Quantifying how much sensory information in a neural code is relevant for behavior. Advances in Neural Information Processing Systems. 2017;30:3686–96.
27. Panzeri S, Senatore R, Montemurro MA, Petersen RS. Correcting for the sampling bias problem in spike train information measures. Journal of neurophysiology. 2007;98(3):1064-72.
28. Optican LM, Gawne TJ, Richmond BJ, Joseph PJ. Unbiased measures of transmitted information and channel capacity from multivariate neuronal data. Biol Cybern. 1991;65(5):305-10. doi: 10.1007/BF00216963. PubMed PMID: 1742368.
29. Strong SP, Koberle R, Van Steveninck RRDR, Bialek W. Entropy and information in neural spike trains. Physical review letters. 1998;80(1):197.
30. Panzeri S, Treves A. Analytical estimates of limited sampling biases in different information measures. Network: Computation in neural systems. 1996;7(1):87-107.
31. Paninski L. Estimation of entropy and mutual information. Neural Computation. 2003;15:1191-253.
32. Kraskov A, Stögbauer H, Grassberger P. Estimating mutual information. Physical Review E. 2004;69(6):066138. doi: 10.1103/PhysRevE.69.066138.
33. Holmes CM, Nemenman I. Estimation of mutual information for real-valued data with error bars and controlled bias. Physical Review E. 2019;100(2):022404. doi: 10.1103/PhysRevE.100.022404.
34. Nemenman I, Bialek W, de Ruyter van Steveninck R. Entropy and information in neural spike trains: Progress on the sampling problem. Physical Review E. 2004;69(5):056111. doi: 10.1103/PhysRevE.69.056111.
35. Chang C-C, Lin C-J. LIBSVM: a library for support vector machines. ACM transactions on intelligent systems and technology (TIST). 2011;2(3):1-27.
36. Friedman JH, Hastie T, Tibshirani R. Regularization Paths for Generalized Linear Models via Coordinate Descent. Journal of Statistical Software. 2010;33(1):1-22. doi: 10.18637/jss.v033.i01.
37. Panzeri S, Moroni M, Safaai H, Harvey CD. The structures and functions of correlations in neural population codes. Nat Rev Neurosci. 2022;23(9):551-67. doi: 10.1038/s41583-022-00606-4. PubMed PMID: 35732917.

38. Averbeck BB, Latham PE, Pouget A. Neural correlations, population coding and computation. Nature Reviews Neuroscience. 2006;7(5):358-66. doi: 10.1038/nrn1888.
39. Rousselet GA, Ince RA, van Rijsbergen NJ, Schyns PG. Eye coding mechanisms in early human face event-related potentials. J Vis. 2014;14(13):7. doi: 10.1167/14.13.7. PubMed PMID: 25385898.
40. Ince RAA, Jaworska K, Gross J, Panzeri S, van Rijsbergen NJ, Rousselet GA, et al. The Deceptively Simple N170 Reflects Network Information Processing Mechanisms Involving Visual Feature Coding and Transfer Across Hemispheres. Cereb Cortex. 2016;26(11):4123-35. doi: 10.1093/cercor/bhw196. PubMed PMID: 27550865.
41. Curreli S, Bonato J, Romanzi S, Panzeri S, Fellin T. Complementary encoding of spatial information in hippocampal astrocytes. PLoS Biol. 2022;20(3):e3001530. doi: 10.1371/journal.pbio.3001530. PubMed PMID: 35239646.
42. Francis NA, Mukherjee S, Kocillari L, Panzeri S, Babadi B, Kanold PO. Sequential transmission of task-relevant information in cortical neuronal networks. Cell Rep. 2022;39(9):110878. doi: 10.1016/j.celrep.2022.110878. PubMed PMID: 35649366.
