# Supplementary material for: MINT: A toolbox for the analysis of multivariate neural information coding and transmission
Source: PLoS Comput Biol. 2025 Apr 15;21(4):e1012934. doi: 10.1371/journal.pcbi.1012934 (PMC12043240; doi:10.1371/journal.pcbi.1012934)
Supplement: Attachment — Submitted filename: Rebuttal Resubmitted.docx [file pcbi.1012934.s004.docx]

**Rebuttal Letter with list of changes for paper PCOMPBIOL-D-24-01933**

Dear Dr. Panzeri,

Thank you for submitting your manuscript to PLOS Computational Biology. After careful consideration, we feel that it has merit but does not fully meet PLOS Computational Biology's publication criteria as it currently stands. Therefore, we invite you to submit a revised version of the manuscript that addresses the points raised during the review process.

Please submit your revised manuscript within 30 days Feb 11 2025 11:59PM. If you will need more time than this to complete your revisions, please reply to this message or contact the journal office at ploscompbiol@plos.org. When you're ready to submit your revision, log on to https://www.editorialmanager.com/pcompbiol/ and select the 'Submissions Needing Revision' folder to locate your manuscript file.

Please include the following items when submitting your revised manuscript:

* A rebuttal letter that responds to each point raised by the editor and reviewer(s). You should upload this letter as a separate file labeled 'Response to Reviewers'. This file does not need to include responses to formatting updates and technical items listed in the 'Journal Requirements' section below.

* A marked-up copy of your manuscript that highlights changes made to the original version. You should upload this as a separate file labeled 'Revised Manuscript with Track Changes'.

* An unmarked version of your revised paper without tracked changes. You should upload this as a separate file labeled 'Manuscript'.

If you would like to make changes to your financial disclosure, competing interests statement, or data availability statement, please make these updates within the submission form at the time of resubmission. Guidelines for resubmitting your figure files are available below the reviewer comments at the end of this letter.

We look forward to receiving your revised manuscript.

Kind regards,

Daniele Marinazzo

Section Editor

PLOS Computational Biology

Additional Editor Comments:

As suggested by one of the reviewers, some pointers to other estimators and implementations would be beneficial.

We added to the toolbox functions computing the KSG and the NSB estimators, as suggested by Reviewers 2 and 3, respectively. These estimators are cited and described in the revised manuscript.

Being this a software paper, a mention of a couple of other toolboxes implementing information theoretical measures, one in matlab https://journals.plos.org/plosone/article?id=10.1371/journal.pone.0109462, and one in python https://joss.theoj.org/papers/10.21105/joss.07360, would help the reader in the navigation of the state of the art.

One of these two references was already cited in the previous submission and it is still cited in the revised manuscript. We added a citation of the second suggested paper, as well as the citations of other papers that will help navigation through the state of the art (see also suggestions of the Reviewers about which methods and papers to add).

Journal Requirements:

1) We ask that a manuscript source file is provided at Revision. Please upload your manuscript file as a .doc, .docx, .rtf or .tex. If you are providing a .tex file, please upload it under the item type u2018LaTeX Source Fileu2019 and leave your .pdf version as the item type u2018Manuscriptu2019.

We uploaded the manuscript source Word (.docx) files.

2) Please provide an Author Summary. This should appear in your manuscript between the Abstract (if applicable) and the Introduction, and should be 150-200 words long. The aim should be to make your findings accessible to a wide audience that includes both scientists and non-scientists. Sample summaries can be found on our website under Submission Guidelines:

<https://journals.plos.org/ploscompbiol/s/submission-guidelines#loc-parts-of-a-submission>

We added the author summary.

3) Please upload all main figures as separate Figure files in .tif or .eps format. For more information about how to convert and format your figure files please see our guidelines:

<https://journals.plos.org/ploscompbiol/s/figures>

We uploaded all figure files in tiff format. We checked that the figures adhere the guidelines and verified it by uploading them to the PACE tool.

4) We have noticed that you have uploaded Supporting Information files, but you have not included a list of legends. Please add a full list of legends for your Supporting Information files after the references list.

We added a list of legends for the Supporting Information files at the end of the document.

5) Figures: 4E, and S6a, include image of an identifiable person. Please provide written confirmation or release forms, signed by the subject(s) (or their guardian), giving permission to be photographed and to have their images published under a Creative Commons license. You may upload permission forms to your submission file inventory as item type 'Other'. Otherwise, we kindly request that you remove the photograph.

We changed the cartoon of Fig 4E and S6A (now Fig F panel A) to replace the photograph of a person with an open-source sketch of a generic, non-identifiable human face. Credits of the open-source sketch are given in the revised text in each relevant figure caption.

6) Some material included in your submission may be copyrighted. According to PLOSu2019s copyright policy, authors who use figures or other material (e.g., graphics, clipart, maps) from another author or copyright holder must demonstrate or obtain permission to publish this material under the Creative Commons Attribution 4.0 International (CC BY 4.0) License used by PLOS journals. Please closely review the details of PLOSu2019s copyright requirements here: PLOS Licenses and Copyright. If you need to request permissions from a copyright holder, you may use PLOS's Copyright Content Permission form.

Please respond directly to this email and provide any known details concerning your material's license terms and permissions required for reuse, even if you have not yet obtained copyright permissions or are unsure of your material's copyright compatibility. Once you have responded and addressed all other outstanding technical requirements, you may resubmit your manuscript within Editorial Manager.

Potential Copyright Issues:

- Figures: 1, 2, and 3; Please confirm whether you drew the images / clip-art within the figure panels by hand. If you did not draw the images, please provide a link to the source of the images or icons and their license / terms of use; or written permission from the copyright holder to publish the images or icons under our CC BY 4.0 license. Alternatively, you may replace the images with open source alternatives. See these open source resources you may use to replace images / clip-art:

- https://commons.wikimedia.org

- https://openclipart.org/.

We wrote to the Editorial Office prior to submission, as requested.

We revised all figures to make sure that every image included in the main text and supplemental figures of the revised paper were either figures hand-drawn by the authors and not used in previous publications or open-source images. In the revised version we credited all open-source resources in each figure caption.

In particular, in the revised figures:

Figure 1: Open-source mouse sketch from [doi.org/10.5281/zenodo.3925917](http://doi.org/10.5281/zenodo.3925917). Open-source brain sketch from [doi.org/10.5281/zenodo.3925989](https://doi.org/10.5281/zenodo.3925989). Open-source speakers were modified from [svgrepo.com/svg/506329/speaker-2](http://www.svgrepo.com/svg/506329/speaker-2), under license CC BY 4.0 (creativecommons.org/licenses/by/4.0).

Figure 2C: mouse sketches hand-drawn by the authors specifically for this paper (image never published before).

Figure 2D and 3B: Open-source mouse sketch from [doi.org/10.5281/zenodo.3925985](http://doi.org/10.5281/zenodo.3925985). Open-source speaker was modified from [svgrepo.com/svg/506329/speaker-2](https://www.svgrepo.com/svg/506329/speaker-2), under license CC BY 4.0 (creativecommons.org/licenses/by/4.0). Open-source music notes were modified from [svgrepo.com/svg/458908/sound](http://www.svgrepo.com/svg/458908/sound), under license CC BY 4.0 (creativecommons.org/licenses/by/4.0).

Figure 4 and F: Human face has been replaced by an open-source generic abstracted human face sketch ([svgrepo.com/svg/493087/men-in-their-20s-and-30s-face](https://www.svgrepo.com/svg/493087/men-in-their-20s-and-30s-face)) with no resemblance to any specific human individual, under CC BY 4.0 license (creativecommons.org/licenses/by/4.0). Head outline was modified from doi.org/10.5281/zenodo.3926093, under CC BY 4.0 license (creativecommons.org/licenses/by/4.0).

Figures A-E do not contain any image (only data plots generated by us).

7) Please amend your detailed Financial Disclosure statement. This is published with the article. It must therefore be completed in full sentences and contain the exact wording you wish to be published. Please ensure that the funders and grant numbers match between the Financial Disclosure field and the Funding Information tab in your submission form. Note that the funders must be provided in the same order in both places as well. State what role the funders took in the study. If the funders had no role in your study, please state: "The funders had no role in study design, data collection and analysis, decision to publish, or preparation of the manuscript.".

If you did not receive any funding for this study, please simply state: u201cThe authors received no specific funding for this work.u201d

We updated the “Funding” and “Competing interests” statements as requested.

Reviewers' comments:

Reviewer's Responses to Questions

Comments to the Authors:

Please note here if the review is uploaded as an attachment.

Reviewer #1: The MINT toolbox is a great resource for computational neuroscientists. Tools for information-theoretic measures in neuroscience exist in a scattered state and often with little documentation or consistency. This toolbox provides an umbrella for a whole suite of applications with consistent syntax and methods. I found the code easy to install on Windows and very well documented.

I only have some very minor comments, and I leave it at the authors’ discretion whether they wish to include them in a revision.

One note is that I did not see mention of embedding for the transfer entropy. Perhaps a brief mention could help? Occasionally, technical concepts are introduced without much explanation. The toolbox covers a lot of ground so this is to some extent inevitable. It also won’t be an obstacle to many users of the toolbox, who come to it for the software tools rather than to learn about the measures themselves. Indeed, the code documentation itself is very helpful in this regard. However, some additional explanation may prove helpful for novice readers who may not yet have a strong conceptual foundation. For example, the use of SVMs could do with a little more elaboration about rationale and implementation. An example of clarification done right is Figure 4A-D, which I found very clear and extremely helpful for following along. The representation of axis misalignment is also very valuable.

We thank the Reviewer for their kind words and for these very useful suggestions about how to improve our paper.

We expanded the justification of the SVM. Following this recommendation and the recommendation of Reviewer 2, we also justify better the choice of SVM as a data dimensionality reduction with new simulations (Fig C in S1 text).

We explain better in the main text and Supplemental Information the delay embedding that is allowed by the toolbox in the TE and FIT measures. The toolbox allows the user to choose an arbitrary user-defined set of time delays, independently for the putative sender and putative receiver. How best to choose these delays is not universally accepted, and different authors have made different choices. We review in Supplemental information some of the constraints and rationale for the choice of embedding delays. In particular, choosing many past time points is good from the theoretical point of view but it makes the estimate high-dimensional and difficult to sample with experimental data. We explain the rules of thumbs for good sampling given the data size. Some authors (especially from the group of Michael Wibral) recommend using delays of one time-step for the past activity as it gives very conservative transferred information estimates. However, in practice and especially with signals such as the EEG that are sampled with a timestep much smaller than the autocorrelation of the signal, using one time step in the past would suppress all information, and using time steps of the order of the autocorrelation length may be recommended. Our own recommendation is to consider and plot a wide range of delays, as we do in our analyses (see e.g. Fig E Panel B in S1 Appendix) to get a better feeling of the robustness of the results as a function of these parameters.

This toolbox will be of great value to the community and I wish to thank the authors for the hard work that clearly went into making and sharing this resource.

Andrea Luppi

Reviewer #2: Review of “MINT: a toolbox for the analysis of multivariate neural information coding and Transmission” by GM Lorenz et al.

This paper describes a toolbox which is in my view an extremely important to quantitative neuroscience. There has been continuing interest, over the years, in information analysis of neurophysiological datasets, with consequent demands for toolboxes to compute these quantities. More recently, there has been substantial interest in computing some slightly more complicated information theoretic quantities, due in particular to the popularisation of partial information decomposition. The challenge is that these quantities can be more difficult to estimate, with at least one widely used public estimation toolbox falling back on “plugin” estimation of entropy – meaning that scientific uses of these tools are likely subject to major pitfalls. The MINT toolbox, which implements a number of advanced information theoretic quantities while at the same time using well-tested and efficient estimators of entropy as their basis, is thus extremely welcome.

The package is demonstrated with application to a diverse set of interesting real world and simulated examples. Some of these examples, however, might better other estimation approaches than those adopted here (see specific points below) and thus I recommend a few small additions to the toolbox in order to make it of greater applicability to a wider range of potential neuroscience users.

We thank the Reviewer for these kind comments and for the suggestions provided to help us improving our work.

Specific points

• The relatively limited number of estimators provided may limit the applicability of the toolbox. For instance, the Kraskov estimator has been of widespread use in the community for information calculation, is one of the best performing estimators for continuous data (see Holmes & Nemenman PRE 20189 for a good discussion), and in particular is used by many for PID calculations. The choice of which estimator to use will depend upon the nature of the dataset analysed – for instance continuous vs discrete/spiking, and in the latter case depending on sparsity of activity. While there are several estimators provided, they are mostly of rather niche utility (eg Ish) and make a rather electic set. I suggest that the authors strongly consider implementing the Kraskov (KSG) estimator (ideally the improved version due to Holmes & Nemenman) alongside the direct method estimation currently present, in order to broaden the utility of the package from niche to general use. And certainly the paper needs a (much) more detailed discussion of entropy and information quantity estimation in the main text, including the provision of advice on when different estimation approaches may be useful.

We now implemented in our revised MINT toolbox the KSG estimator, specifically using the improved version of Holmes and Nemenman (now cited) as suggested by this Reviewer.

We also implemented another impactful estimator, the Nemenman Shafee Bialek (NSB) estimator (now cited), which was recommended by Reviewer 3 and which we found in previous work to be excellent for Mutual Information calculations from simulated spike counts of neuronal populations (Panzeri et al J Neurophysiology 2017).

The KSG and NSB estimators were developed so far only for Shannon information quantities (Entropy, Mutual Information) and thus can be used for Entropy, Mutual Information, Transfer Entropy, and for the versions of PID that are directly computable in terms of Shannon information quantities (e.g., MMI). They cannot be used for other PID that do not rely on Shannon information quantities such as the BROJA or the original formulation of Williams and Beer, which are applicable only to discrete or discretized data. Please note that, because one of the strengths of MINT is the large number of different information theoretic quantities that are implemented, in the paper we focused the application examples on cases in which multiple types of analyses are useful to better address a scientific question. The choice of discrete estimation for these examples is required by the need to use in each application example at least one advanced PID quantities to best address the neuroscientific question posed.

We revised the text (p. 5 and p. 11) to clarify that, with these additions, now the toolbox goes beyond discrete estimation only, including a number further estimators of information all characterized by not relying on parametric models (e.g. Gaussian, Poisson) of the probability distributions, such as the KSG. Estimators relying on non-parametric probability models are good for the cases in which the statistics of the data in unclear or not know. This applies to the majority of current brain experiments, and to essentially all experiments investigating responses of neurons in experimental situations or brain regions not tested before. We have however added among the planned further developments listed on p. 11 the addition of estimators based on parametric probability models.

A complete study of which algorithm would be best suited to each situation is beyond the scope of our paper. We refer to the original publications that investigated these issues extensively. We however expanded the presentation on pages 6-10 with comments on which method we would apply in different conditions, based on considering the simulations performed in this paper and in previous ones, on distilling the literature and on our own experience.

• The tools for dimensionality reduction using decoders is a feature which is absent in most information toolboxes, so this is good to see. It is not clear to me how this interplays with choice of information estimation strategy, and this should be discussed. For instance, I would a priori expect that the discrete estimation strategies adopted here might be suboptimal in these circumstances, and the improved KSG estimator (Holmes & Nemenman) might be best in these circumstances? I am happy to be proved wrong, but I think the paper should provide some validation that the approach is the best one here. The paper demonstrates that it works on the example provided – but you can always find a harder / higher dimensional example, and it would be good to see that it works better than other approaches.

The paper of Holmes and Nemenman, now cited in our revised manuscript, reports a study of the performance of the KSG algorithm for real valued signals and a handful of data dimensions, in which case it works very well. This finding is cited in our revised manuscript (SM 6.4, p. 21 of Supplemental Information).

In the revision of our paper, we added the performance of the estimation algorithms and for stimulus-informative Poisson spike counts of populations of neurons, considering population sizes from N=1 to N=4 for which the ground-truth value of the information carried by the population can be computed numerically with high accuracy (SM 6.4, p. 21 of Supplemental Information).

This allowed us to distinguish two between types of bias. The first is the (usually upward) limited sampling bias, which is the difference between the value of information obtained with the considered neural activity probability estimation method (e.g. response binning, KSG, etc.) when considering a finite number of trials vs when considering an infinite number of trials. The second is the data-processing bias, which reflects the possible inability of the estimation method to process or represent the probability distributions of the data correctly. The data-processing bias can be computed as the difference between the ground truth value of information and the one obtained with the considered estimation method if the data were infinite. For example, when discretizing neural responses into a more limited number of possible discrete responses than the ones that could actually happen (for example, binarizing as all/none the output of a Poisson count), the data estimation bias would be negative.

When using the discrete estimators with binned responses, we found that it had a negligible data-processing bias. The discrete information estimator, without bias correction is however upward biased for large numbers of trials. We tested some of the most used bias correction methods for the discrete information estimators, all implemented in MINT. These bias corrections algorithms worked well and make the estimate convergence close to the ground truth value even for relatively number of trials. In contrast, the KSG has a smaller limited sampling bias but at a cost of a large downward data-processing bias, which reflects the fact that its assumption does not suit discrete data as well as real valued data. We thus would not use it for computing information from neural spike counts. (See SM 6.4, p. 22 of Supplemental Information.

We addressed the interplay between data dimensionality reduction and limited sampling bias in the revised paper by adding (for the N=20 simulated neurons) simulations of different dimensionality reduction algorithms reducing data to 1 dimension. We show (Figure C of Supplemental Information SM 6.4) that in such case the limited sampling bias is well controlled for and that most algorithms extract similar amounts of information even with little training data when information is encoded in very easily detectable features of neural activity (e.g. clear rate separations) while more sophisticated algorithms (e.g. non-linear supervised decoders) and more training data are needed when information is hidden only in more subtle features of neural activity such as the correlations between their activity. We hope that these examples will help the reader to form an intuition about the complex interplay between dimensionality reduction methods and other factors such as number of trials, limited sampling bias corrections and the nature of information encoding. (See SM 6.4, p. 22 of Supplemental Information).

• To extract stimulus-related information, the toolbox uses differences between interaction information in two conditions (stimulus present vs absent or correlated vs independent). However, II is a non-directed information measure that captures the overall synergy between the groups of variables. Therefore, it necessarily requires the user to take a difference with a control condition to isolate the synergy/redundancy the user cares about. In the absence of such controls, the toolbox provides PID measures to extract directed synergy/redundancy. However, a directed version of II known as the Redundancy-Synergy Index (RSI) can provide the benefit of not assuming a redundancy function while characterizing the balance between synergy and redundancy of the system. We suggest implementing RSI as a logical suggestion for completeness, but not a necessity.

We indeed computed RSI to address this question, although we called it with the less well-known name of co-information. We revised the text to now use the RSI nomenclature in both the text and the toolbox function calls. We also note that the Reviewer may have confused our shorthand II (that we used for Intersection Information) as Interaction Information. We revised the paper’s main text to spelling out more often in full “Intersection Information”.

• The toolbox aims to be universally applicable to different modalities of data (EEG,fMRI,Spiking etc), by relying on discretisation of the signal. Then, using discrete information estimators. This method although useful in information-theoretic estimation of continuous signals has many limitations. Especially when the temporal structure of continuous signals may be implicated. Different binning protocols may be useful for different kinds of analysis. Such limitations should be discussed especially in case of continuous signals such as M/EEG and fMRI.

Thanks to the new additions of estimation methods suggested by Reviewer 2 and 3, the toolbox also implements other useful methods beyond discretization, including those for quantification of information transfer (TE) using continuous valued algorithms that the Reviewer alludes to. Thus, the specific limitation mentioned by the Reviewer has been significantly ameliorated. We have also expanded the discussion other limitations in Discussion. Please note that software articles are required to be short, and upon consultation with the Editor we have decided to keep all main text revisions as concise as possible.

• It is in some ways unfortunate that the toolbox is implemented in MATLAB, as this language is now falling out of use among the computer-literate neuroscience communities (and even engineering communities) that form the target audience. A python implementation would have been likely to find much more widespread use. The authors should comment on how the system could be used from python using for instance the MATLAB Engine API for Python.

We did run the MINT toolbox with MATLAB Engine API for Python as suggested, and it works well. We added to the Supplemental Information (Section SM2, p. 3) a description of how to do it and we added to the software Repository instructions of how to set up the engine and examples scripts on how to run example analyses of MINT from python using this engine.

• Overall, limitations of the toolbox, i.e. under what conditions it is not applicable, should be discussed. I would suggest a longer discussion section in which advice is provided about what the toolbox can do well in its current form, what it can do but may not be ideally configured for, and what still needs to be implemented.

We have expanded Discussion with more descriptions of the limitations. Because of the tight word count limit, we kept the Discussion short to meet the word count agreed with the Editor. Also, the PCB Software Paper instructions state that the Discussion should be focused on the future additions to the code in specific, so our text about limitations is mostly framed as features that the toolbox does not have and that we plan or wish to add.

• Figure 1, panel E, “plague” is exactly how it feels, but I suggest the choice of a less loaded term.

We removed from the caption of Figure 1 the part of the sentence using the word “plague” as it was not necessary.

Very Minor

• Some typos, eg “empirically occurrences” (p. 4). The manuscript would benefit from a pass to correct these.

We fixed the mentioned typo and we did our best to fix all other remaining typos or imprecision that we found.

Reviewer #3: In this manuscript, the authors describe a set of computational methods, implemented in MATLAB, for computing a number of information theoretic measures that are relevant to interpreting behavioral neuroscience data. These include measures that can be used on single cells and pairs of cells (including those that look for synergistic encoding and those that measure transfer of information across time), as well as methods for incorporating larger groups of neurons by implementing basic dimensionality reduction, both unsupervised (e.g. PCA) and supervised (e.g. SVM). The authors usefully present a few examples of applying the methods to real and simulated datasets.

The manuscript is for the most part clearly presented, and the existence of such a toolbox will no doubt be beneficial to the field. The lack of a clear consensus in the literature of the most useful measures means that there are other related measures that are not represented here (e.g. other ways of dealing with small-N bias such as the NSB method; other ways of computing synergy among larger sets of neurons such as the O-information)--- but the included set of measures is large enough that this is easily more complete than any other existing software package.

We thank the Reviewer for their kind words about our work and for noting the extensiveness of the set of measures already included.

We added the NSB estimator as suggested by this Reviewer, and we also added the KSG estimators suggested by Reviewer 2. See responses to Reviewer 2 for more details.

Some relatively minor concerns:

— I was confused by the Fig 3C left plot. Is this just an example of one particular pair of neurons?

Yes, Figure 3C reports an example pair of neurons. This was stated already in the main text and in the Figure caption, but we now added it to the figure panel title to make it more salient.

— In the simulated example in Fig. 4, what is the particular dynamic of “sending” information? E.g. what is the timescale of information transfer, and what needs to be known about these timescales for this to work in analyzing real data?

This is all detailed in the Supplemental Information Section SM6.3. We added a sentence to the main text (page 9) to summarize these facts.

— A few typos: "will lead to uncover numerous new insights"; "as empirically occurrences"; "directly form"

Fixed, thanks.

Figure resubmission:

While revising your submission, please upload your figure files to the Preflight Analysis and Conversion Engine (PACE) digital diagnostic tool, https://pacev2.apexcovantage.com/. PACE helps ensure that figures meet PLOS requirements. To use PACE, you must first register as a user. Registration is free. Then, login and navigate to the UPLOAD tab, where you will find detailed instructions on how to use the tool. If you encounter any issues or have any questions when using PACE, please email PLOS at figures@plos.org. Please note that Supporting Information files do not need this step. If there are other versions of figure files still present in your submission file inventory at resubmission, please replace them with the PACE-processed versions.

We checked figures with PACE as suggested.

Reproducibility:

To enhance the reproducibility of your results, we recommend that authors of applicable studies deposit laboratory protocols in protocols.io, where a protocol can be assigned its own identifier (DOI) such that it can be cited independently in the future. Additionally, PLOS ONE offers an option to publish peer-reviewed clinical study protocols. Read more information on sharing protocols at <https://plos.org/protocols?utm_medium=editorial-email&utm_source=authorletters&utm_campaign=protocols>

We decided not register the protocol, as it does not seem very helpful in this case.
